# The Human Adenovirus E4-ORF1 Protein Subverts Discs Large 1 to Mediate Membrane Recruitment and Dysregulation of Phosphatidylinositol 3-Kinase

**DOI:** 10.1371/journal.ppat.1004102

**Published:** 2014-05-01

**Authors:** Kathleen Kong, Manish Kumar, Midori Taruishi, Ronald T. Javier

**Affiliations:** Department of Molecular Virology and Microbiology, Baylor College of Medicine, Houston, Texas, United States of America; University of Michigan, United States of America

## Abstract

Adenoviruses infect epithelial cells lining mucous membranes to cause acute diseases in people. They are also utilized as vectors for vaccination and for gene and cancer therapy, as well as tools to discover mechanisms of cancer due to their tumorigenic potential in experimental animals. The adenovirus *E4-ORF1* gene encodes an oncoprotein that promotes viral replication, cell survival, and transformation by activating phosphatidylinositol 3-kinase (PI3K). While the mechanism of activation is not understood, this function depends on a complex formed between E4-ORF1 and the membrane-associated cellular PDZ protein Discs Large 1 (Dlg1), a common viral target having both tumor suppressor and oncogenic functions. Here, we report that in human epithelial cells, E4-ORF1 interacts with the regulatory and catalytic subunits of PI3K and elevates their levels. Like PI3K activation, PI3K protein elevation by E4-ORF1 requires Dlg1. We further show that Dlg1, E4-ORF1, and PI3K form a ternary complex at the plasma membrane. At this site, Dlg1 also co-localizes with the activated PI3K effector protein Akt, indicating that the ternary complex mediates PI3K signaling. Signifying the functional importance of the ternary complex, the capacity of E4-ORF1 to induce soft agar growth and focus formation in cells is ablated either by a mutation that prevents E4-ORF1 binding to Dlg1 or by a PI3K inhibitor drug. These results demonstrate that E4-ORF1 interacts with Dlg1 and PI3K to assemble a ternary complex where E4-ORF1 hijacks the Dlg1 oncogenic function to relocate cytoplasmic PI3K to the membrane for constitutive activation. This novel mechanism of Dlg1 subversion by adenovirus to dysregulate PI3K could be used by other pathogenic viruses, such as human papillomavirus, human T-cell leukemia virus type 1, and influenza A virus, which also target Dlg1 and activate PI3K in cells.

## Author Summary

Adenoviruses cause acute illnesses in people, and are additionally utilized both as vehicles to cure genetic diseases, fight cancer, and deliver vaccines, and as tools to discover how cancers develop due to a capacity to generate tumors in experimental animals. The adenovirus E4-ORF1 protein reprograms cell metabolism to enhance virus production in infected cells and promotes cell survival and tumors by activating the important cellular protein phosphatidylinositol 3-kinase (PI3K). How E4-ORF1 activates PI3K is not known, though this function depends on E4-ORF1 binding to the membrane-associated cellular protein Discs Large 1 (Dlg1), which many different viruses evolved to target. In this study, we identify PI3K as a new direct target of E4-ORF1. Results further show that E4-ORF1 binds to PI3K in the cytoplasm and delivers it to Dlg1 at the membrane where the three proteins form a complex that activates PI3K and induces oncogenic growth in cells. This novel molecular mechanism in which adenovirus subverts Dlg1 to dysregulate PI3K may serve as a paradigm to understand PI3K activation mediated by other important pathogenic viruses, such as human papillomavirus, human T-cell leukemia virus type 1, and influenza A virus, which also target Dlg1 in infected cells.

## Introduction

Human adenovirus type 9 (Ad9) is a member of the subgroup D adenoviruses that cause eye infections in people [1]. In addition, infection of experimental animals with Ad9 generates estrogen-dependent mammary tumors, and the *E4-ORF1* gene is the primary viral oncogenic determinant [2]–[4]. This viral gene likely evolved from a cellular *dUTPase* gene, which codes for an enzyme of nucleotide metabolism, and E4-ORF1 and dUTPase share a similar protein fold [5], [6]. However, the E4-ORF1 protein lacks dUTPase catalytic activity, indicating functional divergence from dUTPase. Instead, E4-ORF1 functions to activate cellular class IA phosphatidylinositol 3-kinase (PI3K) at the plasma membrane of Ad9-infected human epithelial cells and Ad9-induced experimental tumor cells [7]. This function is conserved in other human adenovirus E4-ORF1 proteins and is essential for Ad9-induced oncogenesis [7]. E4-ORF1 activation of PI3K also enhances productive replication of human adenovirus type 5 (Ad5) by overriding protein translation checkpoints [8], [9], prolongs survival of Ad5 vector-infected primary human endothelial cells [10], and modulates lipid and glucose metabolism in human adenovirus type 36-infected cells [11].

Class IA PI3K is a lipid kinase that under normal physiological conditions functions as a key downstream effector of membrane receptors and ras [12]. PI3K exists as a heterodimer composed of p85 regulatory and p110 catalytic subunits. In the cytoplasm, the regulatory subunit stabilizes the catalytic subunit and inhibits its lipid kinase activity. Activated membrane receptors and ras can bind and recruit cytoplasmic PI3K to the plasma membrane, bringing it into contact with the lipid substrate phosphatidylinositol-4,5-bisphosphate (PIP2) and also relieving enzymatic inhibition by the p85 regulatory subunit. PI3K converts PIP2 to the second messenger phosphatidylinositol 3,4,5-trisphosphate (PIP3), which in turn recruits PI3K effector proteins Akt and PDK1 to the plasma membrane. At this site, Akt is activated by phosphorylation on threonine 308 (T308) by PDK1 and on serine 473 (S473) by mTORC2. Numerous downstream effectors of Akt act to regulate a broad range of cellular processes that include metabolism, protein synthesis, growth, survival, migration, and proliferation. Notably, the PI3K signaling pathway is one of the most frequently dysregulated pathways in human cancers [13], and PI3K and its downstream effectors are subverted by many pathogenic human viruses to enhance virus-cell and virus-host interactions, such as viral entry, replication, reactivation from latency, and pathogenesis [14]. These observations underscore a prominent role for the PI3K pathway in human disease.

Mutational analyses of the 125-residue Ad9 E4-ORF1 polypeptide identified multiple amino acid residues required for activation of PI3K, promotion of oncogenic transformation in cultured cells, and tumorigenesis in experimental animals [7], [15]–[17]. Some of these crucial residues cluster at the E4-ORF1 carboxyl-terminus [18], which defines an element that mediates direct binding to cellular PSD95/Dlg/ZO-1 (PDZ) domain proteins [19], thereby revealing the first known virus-encoded PDZ domain-binding motif (PBM). The identification of the E4-ORF1 PBM led to the discovery that the E6 and Tax proteins encoded by high-risk HPV or HTLV-1, respectively, also possess a carboxyl-terminal PBM that is crucial for their oncogenic potential [19]–[24]. It is now recognized that many viruses code for PBM-containing proteins that target a wide variety of cellular PDZ proteins [23].

Dlg1 was the first cellular PDZ protein reported to interact with the PBM of Ad9 E4-ORF1, as well as HPV E6 and HTLV-1 Tax [19]. Subsequent studies showed that the E4-ORF1 PBM also mediates binding to the cellular PDZ proteins MUPP1, PATJ, MAGI-1, and ZO-2 [25]–[28]. In polarized epithelial cells, Dlg1 localizes to the adherens junction (AJ), whereas MUPP1, PATJ, MAGI-1, and ZO-2 localize to the tight junction (TJ). These PDZ proteins normally function as scaffolds to assemble plasma membrane-associated protein complexes that control signal transduction, AJ and TJ formation, and polarity establishment [22], [23].

In cells, E4-ORF1 exists as both a monomer and homo-trimer, each of which targets a different subset of PDZ proteins with distinct functional outcomes [5]. The E4-ORF1 monomer specifically binds and sequesters MUPP1, PATJ, MAGI-1, and ZO-2 within cytoplasmic punctae [25], [26], [28] and, as a result, disrupts the TJ and causes a loss of cell polarity [27]. Such defects are hallmarks of cancer cells and may directly contribute to carcinogenesis by deregulating normal cellular proliferation and differentiation programs [29]–[31]. More relevant to the current study, the E4-ORF1 trimer specifically binds to Dlg1 [5], which consists of three PDZ domains along with Lin-2 and Lin-7 (L27), Src homology 3 (SH3), and guanylate kinase-homology (GuK) domains, as well as different insertion elements (I1-I5) generated by alternative splicing [32]. While evidence indicates that Dlg1 has a tumor suppressor function [33], *Dlg1^+/+^* but not mutant *Dlg1* ^−/−^ mouse embryo fibroblasts are able to support oncogenic PI3K activation by Ad9 E4-ORF1 [34]. This finding not only revealed an absolute dependence on Dlg1 for this activity but also exposed a previously unrecognized Dlg1 oncogenic function, which may be widely important given that high-risk HPV E6 proteins require Dlg1 to promote invasive properties in cervical carcinoma cells [33], [35]. The Dlg1 oncogenic function hijacked by E4-ORF1 derives from a specific Dlg1 splice isoform, Dlg1-I3, which has an I3 insertion element that localizes Dlg1 to the plasma membrane by binding to membrane-associated protein 4.1 [36], [37]. Moreover, Dlg1-I3 contributes to E4-ORF1-mediated PI3K activation, at least in part, by recruiting E4-ORF1 to the plasma membrane [34], implying that the Dlg1:E4-ORF1 complex activates PI3K at this site. However, the molecular mechanism of activation has not been determined and remains an important gap in knowledge.

The present study was undertaken to test the hypothesis that PI3K activation depends on an additional undetermined cellular factor recruited by E4-ORF1 into the Dlg1:E4-ORF1 complex. This idea prompted a search for new E4-ORF1-interacting proteins and led to identification of PI3K itself. Our findings revealed that E4-ORF1 binds directly to PI3K and recruits it into the Dlg1:E4-ORF1 complex, thereby forming a Dlg1:E4-ORF1:PI3K ternary complex that localizes to the plasma membrane and stimulates PI3K signaling. This novel mechanism of PI3K activation may serve as a paradigm to understand how other pathogenic human viruses dysregulate PI3K and how the common viral target Dlg1, and possibly other PDZ proteins, contributes to viral infections and diseases.

## Results

### Cellular PI3K is a direct target for the adenovirus E4-ORF1 protein

**Figure 1:**
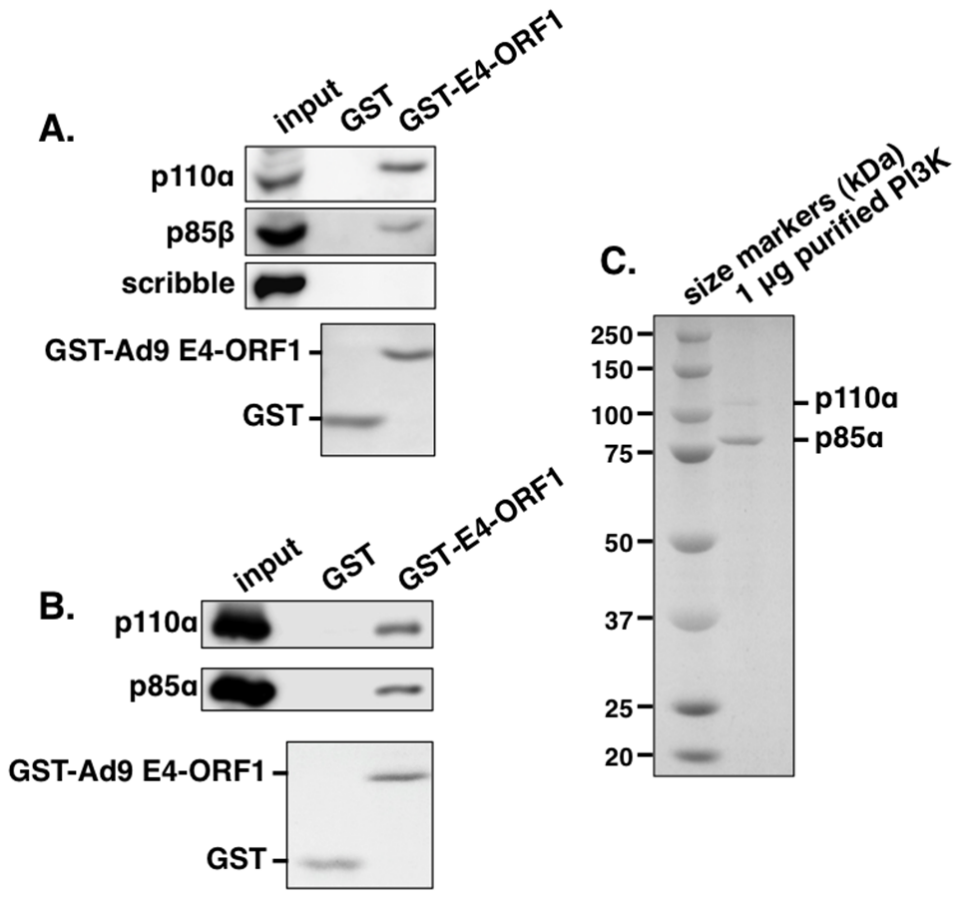
The GST-E4-ORF1 fusion protein binds specifically and directly to the functional PI3K p85:p110 heterodimer in an *in vitro* pulldown assay. (**A**) The GST-E4-ORF1 protein binds to the endogenously expressed p85 regulatory and p110 catalytic PI3K subunits in MCF10A cell lysates. MCF10A cell extract (500 µg of protein) was subjected to pulldown assays with the indicated GST fusion protein. Recovered proteins, as well as cell extract (input), were analyzed in an immunoblot assay with antibodies to the indicated proteins. Coomassie Brilliant Blue staining of the lower portion of the protein gel verified use of an equivalent amount of GST and GST-Ad9 E4-ORF1 protein. (**B**) The GST-E4-ORF1 protein also binds to purified, functional recombinant human PI3K. Purified, recombinant human PI3K protein (1 µg; >90% pure full-length p85α and p110α) (see *Materials and Methods*) was subjected to a pulldown assay with either control GST or GST-Ad9 E4-ORF1 protein. Recovered proteins and 1/2 of input PI3K protein were analyzed in an immunoblot assay with p85α and p110α antibodies. Coomassie Brilliant Blue staining of the lower portion of the protein gel verified use of an equivalent amount of GST and GST-Ad9 E4-ORF1 protein in the assay. (**C**) Verification of the purity of the recombinant PI3K protein. The input PI3K protein (1 µg) used in Figure 1B was resolved by SDS-PAGE, and the protein gel was stained with Coomassie Brilliant Blue. The recombinant p85 protein is present in slight molar excess over the recombinant p110 protein in the purified PI3K preparation.

To identify the postulated cellular factor that binds to E4-ORF1 in the Dlg1:E4-ORF1 complex, we subjected HeLa cell lysates to an *in vitro* pulldown assay with glutathione *S*-transferase (GST) fused to Ad9 E4-ORF1, followed by a mass spectrometry analysis of associated proteins. **Table S1** lists 10 selected identified proteins, eight of which were either identical (Abl1, ELAV1, IGF2BP1, IGF2BP3) or related to (UPF2, LARP1, LARP2, DDX17) Ad5 E4-ORF1-binding proteins reported in a recent proteomic study of DNA tumor virus oncoproteins [38], suggesting that these eight proteins may be genuine E4-ORF1 cellular targets. More importantly, the other two proteins in the list were novel, and their identities as the PI3K regulatory subunits p85α and p85β prompted additional experiments to confirm and extend the finding. We first used immunoblots to examine proteins recovered from a similar pulldown conducted with lysates of human MCF10A cells, an immortalized but non-transformed human mammary epithelial cell line retaining properties of normal breast epithelial cells [39]. The results showed that GST-E4-ORF1 but not GST interacts with p85β and the PI3K catalytic subunit p110α and that neither GST protein associates with the cellular PDZ protein scribble (**Figure 1A**). Moreover, GST-E4-ORF1 but not GST also interacted with the purified, catalytically-active recombinant human p85α:p110α heterodimer (**Figures 1B and 1C**). The low binding efficiency of GST-E4-ORF1 seen in these assays is a general property attributed to aggregation of this bacterially expressed fusion protein. These collective data indicated specific and direct binding of Ad9 E4-ORF1 to functional class IA human PI3K.

### E4-ORF1 mediates Dlg1-dependent PI3K activation and protein elevation in human epithelial cells

To investigate whether E4-ORF1 associates with endogenous PI3K in human epithelial cells, we generated and characterized MCF10A lines transduced either by the empty pBABE retroviral expression vector (vector cells) or by pBABE encoding the *wild-type* (*wt*) Ad9 E4-ORF1 protein (*wt*ORF1 cells), the Ad9 E4-ORF1 PBM mutant V125A protein unable to bind PDZ proteins (V125A cells), or the mutant rasV12 protein that lacks a PBM but activates PI3K and the Raf/MAP kinase pathway (rasV12 cells) [7], [19].

**Figure 2:**
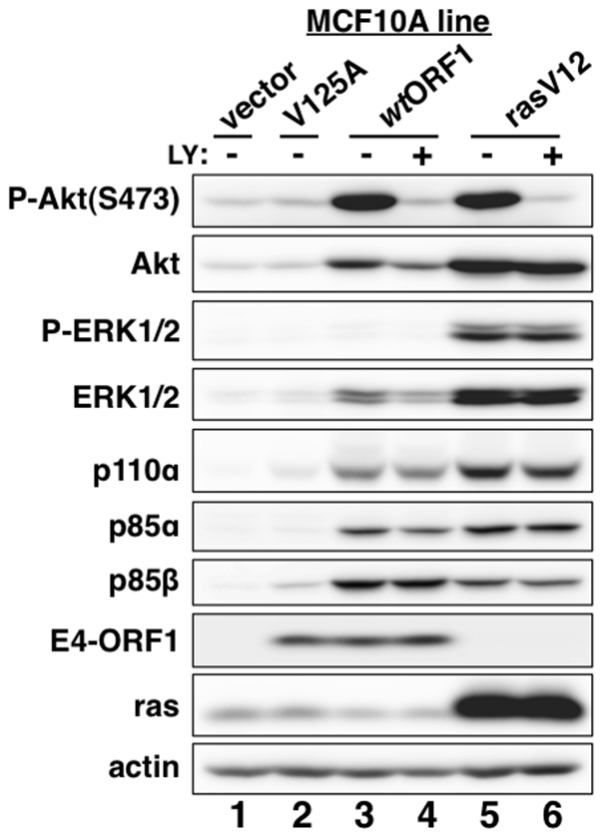
E4-ORF1 activates PI3K and upregulates PI3K protein levels in a PBM-dependent manner but does not activate the MAP kinases ERK1 and ERK2. MCF10A lines transduced with empty retroviral vector (vector cells) or the vector encoding *wt* Ad9 E4-ORF1 (*wt*ORF1 cells), PBM mutant Ad9 E4-ORF1 V125A (V125A cells), or rasV12 (rasV12 cells) were treated with either DMSO vehicle (−) or 100 µM LY294002 (LY) (+) for 30 min. Cell extracts were analyzed in an immunoblot assay with antibodies to the indicated proteins.

**Figure 3:**
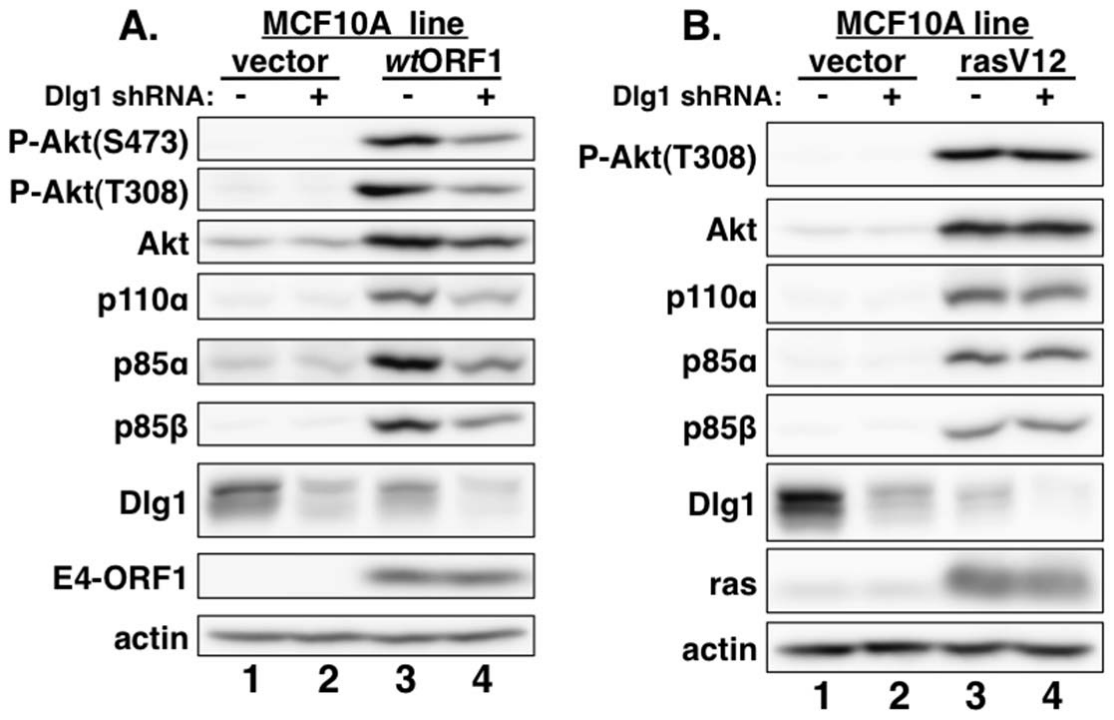
E4-ORF1 activates PI3K and upregulates PI3K protein levels in a Dlg1-dependent manner. (**A**) Depletion of Dlg1 with an shRNA diminishes PI3K activation and PI3K protein elevation in *wt*ORF1 cells. Extracts from vector or *wt*ORF1 cells stably transduced with pSUPER-Dlg1 shRNA (+) or pSUPER-Dlg1 scrambled control shRNA (−) were analyzed in an immunoblot assay with antibodies to the indicated proteins. (**B**) Depletion of Dlg1 has no effect on either PI3K activation or PI3K protein elevation in rasV12 cells. Extracts of rasV12 cells transduced with pSUPER-Dlg1 shRNA (+) or pSUPER-Dlg1 scrambled control shRNA (−) were analyzed in an immunoblot assay with antibodies to the indicated proteins.

Consistent with previous results in rodent fibroblast lines [7], immunoblots of extracts from the cell lines revealed higher levels of activated PI3K effector Akt dually phosphorylated at T308 and S473 (P-Akt) in *wt*ORF1 and rasV12 cells than in vector and V125A cells (**Figure 2**, compare lanes 1–2 to lanes 3 and 5; **Figures 3A and 3B**, compare lane 1 and lane 3), as well as higher levels of activated, phosphorylated MAP kinases ERK-1 and -2 (P-ERK1/2) in rasV12 cells than in other cells (**Figure 2**, compare lane 5 to lanes 1–3) [34]. Compared to vector and V125A cells, w*t*ORF1 and rasV12 cells also displayed higher levels of p85α, p85β, and p110α (**Figure 2**, compare lanes 1–2 to lanes 3 and 5). Moreover, treatment of *wt*ORF1 and rasV12 cells with the PI3K inhibitor drug LY294002 (LY) returned the high P-Akt levels to those of vector cells, yet had little or no effect on the high p85 and p110 levels (**Figure 2**, compare lane 1 to lanes 3–4 and 5–6). In contrast, stable expression of a short hairpin RNA (shRNA) that depleted Dlg1 decreased the high levels of P-Akt, p85, and p110 in *wt*ORF1 cells but not in rasV12 cells (compare **Figure 3A**, lanes 3–4 to **Figure 3B**, lanes 3–4). From cumulative immunoblots, we quantified pertinent protein level differences for *wt*ORF1 or rasV12 cells versus vector cells, as well as for *wt*ORF1 cells transduced with the Dlg1 shRNA vector versus the scrambled shRNA vector (**Tables S2, S3, and S4**). Taken together, the data showed that while E4-ORF1 and rasV12 similarly elevate p85α, p85β, and p110α levels and activate Akt in a PI3K-dependent manner in human epithelial cells, E4-ORF1 differs from rasV12 in its dependence on both a PBM and Dlg1 for these activities and in not activating the Raf/MAP kinase pathway.

### The Dlg1-I3 isoform mediates E4-ORF1-induced PI3K activation whereas both the Dlg1-I3 and -I2 isoforms mediate PI3K protein elevation

The Dlg1-I2 isoform differs from the Dlg1-I3 isoform by having an I2 rather than I3 splice insertion element and by failing to support E4-ORF1-induced PI3K activation in rodent fibroblasts [34]. Given that Dlg1 depletion diminished both PI3K activation and PI3K protein elevation by E4-ORF1 in MCF10A cells (**Figure 3A**), we sought to clarify roles for each Dlg1 isoform in these two E4-ORF1 activities.

**Figure 4:**
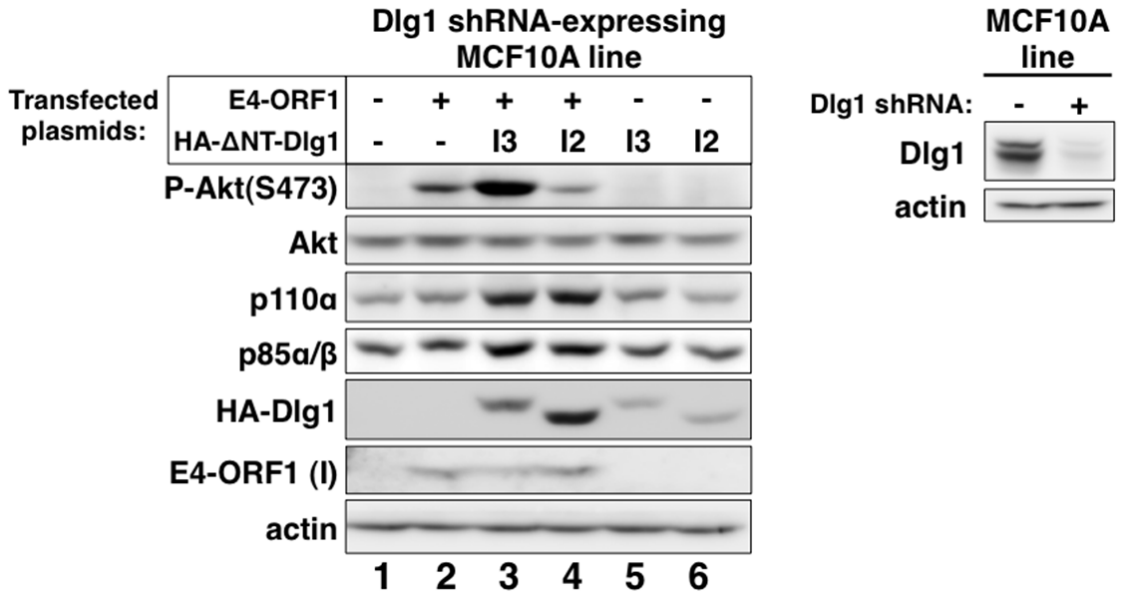
Only Dlg1-I3 mediates E4-ORF1-induced PI3K activation whereas both Dlg1-I3 and Dlg1-I2 mediate PI3K protein elevation. *Left panel*, The Dlg1 shRNA-expressing MCF10A line was transfected with expression plasmid GW1-E4-ORF1 (75 ng), GW1-HA-ΔNT-Dlg1-I3 (2 µg), or GW1-HA-ΔNT-Dlg1-I2 (4 µg) alone or in the indicated combinations. The total amount of DNA in each transfection was equalized to 7.575 µg using empty GW1 plasmid. At 48 h post-transfection, cells were serum starved for 1 h, and then extracts were prepared and analyzed in immunoblot assays using the indicated antibodies. The insoluble (I) pellet fraction (see *Materials and Methods*) was immunoblotted with E4-ORF1 antibody. *Right panel*, This blot verifies Dlg1 protein depletion in the Dlg1 shRNA-expressing MCF10A line (+) used in the left panel by comparison to the control Dlg1 scrambled shRNA-expressing MCF10A line (−).

Our chosen approach was to determine whether reconstitution of Dlg1-depleted MCF10A cells with each Dlg1 isoform restores E4-ORF1-induced PI3K activation and/or PI3K protein elevation. We therefore transfected the Dlg1 shRNA-expressing MCF10A line with a *wt* E4-ORF1 expression plasmid alone or in combination with an expression plasmid encoding HA epitope-tagged (HA-) ΔNT-Dlg1-I3 or ΔNT-Dlg1-I2 (**Figure 4**, *left panel*), both of which have the amino-terminal (NT) region deleted. Deletion of the NT region, which contains the Dlg1 shRNA targeting sequence, renders HA-ΔNT-Dlg1 expression refractory to depletion by the Dlg1 shRNA, but does not affect the ability of Dlg1-I3 to support E4-ORF1-induced PI3K activation [34]. To detect the low levels of E4-ORF1 protein produced in these transient transfection assays, we immunoblotted for E4-ORF1 in the RIPA buffer-insoluble cell pellet fraction, which contains 91%±2.5% (n = 3 independent experiments) of E4-ORF1 protein expressed in MCF10A cells (**Fig. S1**). E4-ORF1 displays a similar cell fractionation profile in rodent fibroblasts [5], [15], [34]. A control immunoblot also verified Dlg1 depletion in the Dlg1 shRNA-expressing MCF10A line (**Figure 4**, *right panel*). The data showed that E4-ORF1-induced PI3K activation is increased by HA-ΔNT-Dlg1-I3 or decreased by HA-ΔNT-Dlg1-I2 and that E4-ORF1-induced p110α and p85α/β protein elevation is enhanced similarly by either HA-ΔNT-Dlg1-I3 or -I2 (**Figure 4**, *left panel*, compare lanes 2–4). From cumulative independent experiments, we quantified these effects induced by Dlg1-I3 (**Table S5**) or Dlg1-I2 (**Table S6**). Based on the findings, we concluded that, in Dlg1 shRNA-expressing MCF10A cells, Dlg1-I3 expression restores E4-ORF1-induced PI3K activation whereas either Dlg1-I3 or Dlg1-I2 expression restores PI3K protein elevation. The latter observation with Dlg1-I2 also demonstrated that E4-ORF1-induced PI3K activation and PI3K protein elevation are distinct Dlg1 activities, and revealed the first known function for the E4-ORF1:Dlg1-I2 complex.

We previously reported that ras is required for E4-ORF1-induced PI3K activation in mouse fibroblasts [34]. To test whether this finding would extend to the current system, we transfected MCF10A cells with a *wt* E4-ORF1 expression plasmid alone or in combination with an expression plasmid encoding dominant-negative mutant rasN17. The data showed that rasN17 over-expression blocked PI3K activation induced by mutant rasv12 (**Figure S2A**, compare lanes 3–4) but not E4-ORF1 (**Figure S2B**, compare lanes 3–4). These findings suggested that ras does not mediate E4-ORF1-induced PI3K activation in MCF10A cells.

### E4-ORF1 assembles a ternary complex that tethers Dlg1 to PI3K in E4-ORF1-expressing and adenovirus-infected human epithelial cells

**Figure 5:**
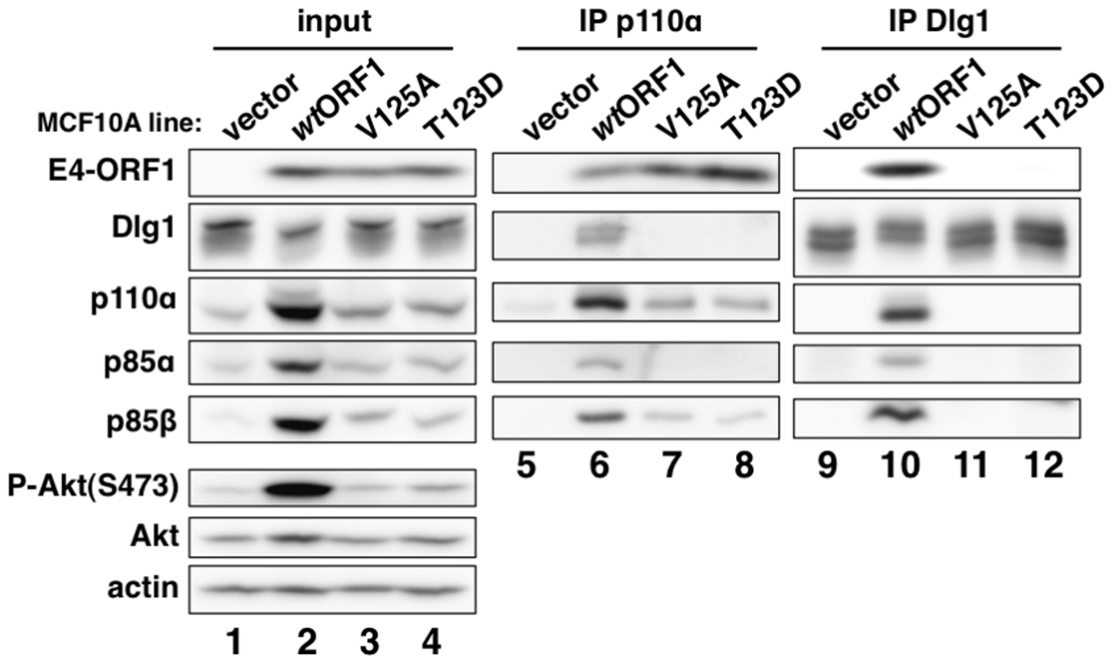
E4-ORF1 binds endogenous cellular PI3K and tethers it to Dlg1 to form a ternary complex in cells. Extracts (300 µg of protein) of vector, *wt*ORF1, V125A, and T123D cells were immunoprecipitated with either p110α antibody or Dlg1 antibody as indicated. Recovered proteins, as well as cell extract (input), were analyzed in an immunoblot assay with antibodies to the indicated proteins. Note that V125A and T123D cells express Ad9 E4-ORF1 PBM mutants unable to bind PDZ proteins.

We next immunoprecipitated (IPed) p110α from lysates of vector, *wt*ORF1, and V125A cells, as well as T123D cells expressing the Ad9 E4-ORF1 PBM mutant T123D protein unable to bind PDZ proteins [7], [19], and then tested for co-immunoprecipitation (CoIP) of E4-ORF1, Dlg1, p85α, and p85β in immunoblots (**Figure 5**). The data showed comparable coIP of *wt* and mutant E4-ORF1 proteins with p110α from each respective cell line (lanes 6–8), and also coIP of Dlg1 with p110α only from *wt*ORF1 cells (compare lane 6 to lanes 7–8). IPed p85α or p85β yielded similar results (data not shown).

**Figure 6:**
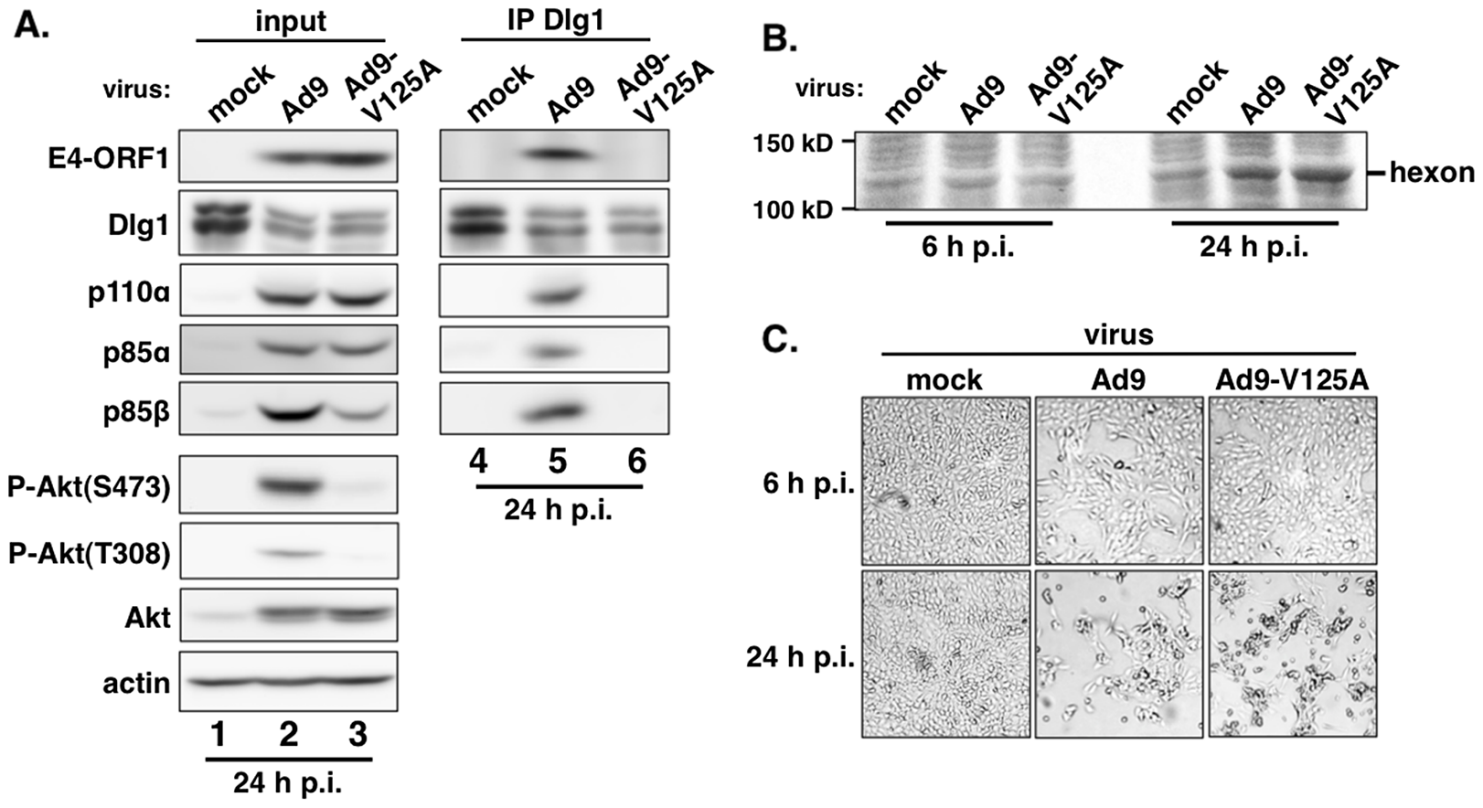
E4-ORF1 also assembles the ternary complex in adenovirus-infected cells. (**A**) The ternary complex forms in cells infected with *wt* Ad9 virus but not with mutant Ad9-V125A virus that expresses PBM mutant V125A. MCF10A cells were mock infected or infected with the indicated virus at an approximate multiplicity of infection of 1. Extracts (375 µg of protein) prepared at 24 h post infection (p.i.) were immunoprecipitated with Dlg1 antibody. Recovered proteins, as well as cell extract (input), were analyzed in an immunoblot assay with antibodies to the indicated proteins. (**B**) Cells that are infected with *wt* Ad9 or mutant Ad9-V125A virus accumulate similar amounts of adenovirus major capsid protein hexon. Cell extracts were resolved by SDS-PAGE and stained with Coomassie Brilliant Blue. (**C**) Cells that are infected with *wt* Ad9 or mutant Ad9-V125A virus display similar cytopathic effects. Cells were visualized by phase contrast microscopy (40× magnification).

In a reciprocal experiment, we IPed Dlg1 from lysates of the same cell lines and tested for coIP of E4-ORF1, p110α, p85α, and p85β (**Figure 5**). Here we observed coIP of the *wt* E4-ORF1 protein but not PBM mutant V125A or T123D protein with Dlg1 as expected, and also co-IP of p110α, p85α, and p85β with Dlg1, but again only from *wt*ORF1 cells (compare lane 10 to lanes 11–12). Significantly, the latter results with vector, *wt*ORF1, and V125A cells were mirrored when Dlg1 was IPed from lysates of mock-infected MCF10A cells or MCF10A cells infected with *wt* Ad9 virus or mutant Ad9 virus having the PBM mutation V125A introduced into the *E4-ORF1* gene (Ad9-V125A) (**Figure 6A**, compare lane 5 to lanes 4 and 6). As controls, we verified that the infection with each virus was comparable by examining E4-ORF1 protein levels (**Figure 6A**), viral major capsid protein hexon accumulation (**Figure 6B**), and cytopathic effects (**Figure 6C**). The comparable hexon protein accumulation was consistent with our failure to detect a replication defect for the Ad9-V125A mutant virus in MCF10A cells (unpublished results). In addition, *wt* Ad9 virus-infected cells showed higher levels of activated P-Akt than either Ad9-V125A virus- or mock-infected cells (**Figure 6A**; **Tables S7 and S8**), similar to previous results examining P-Akt activation in human A549 cells mock-infected or infected with *wt* Ad9 or mutant Ad9-IIIA virus, which like the Ad9-V125A virus encodes an *E4-ORF1* gene with a disrupted PBM [7]. We also observed decreased Dlg1 levels in cells infected with either *wt* Ad9 virus or Ad9-V125A virus compared to mock-infected cells (**Figure 6A**, compare lane 1 and lanes 2–3). A similar effect was detected in rasV12-expressing cells (**Figure 3B**, compare lanes 1 and 3), whereas the modest reduction in Dlg1 levels seen in *wt*ORF1 cells compared to vector cells in **Figure 3A** was not reproducible. We do not yet understand how Dlg1 is downregulated in adenovirus-infected cells and RasV12-expressing cells.

The biochemical data presented above importantly showed that in human epithelial cells, either stably expressing *wt* E4-ORF1 protein or lytically infected with *wt* Ad9 virus, the E4-ORF1 protein binds the PI3K p85:p110 heterodimer in a PBM-*independent* manner and, in conjunction with separate PBM-*dependent* binding to Dlg1, tethers these two cellular factors together to form a Dlg1:E4-ORF1:PI3K ternary complex. Moreover, formation of this ternary complex strictly correlated with E4-ORF1-mediated PI3K/Akt activation as we observed the complex in *wt*ORF1 cells and *wt* Ad9 virus-infected cells but not in V125A cells, T123D cells, or mutant Ad9-V125A virus-infected cells.

Nonetheless, immunoblots of lysates from *wt* Ad9 virus- and mutant Ad9-V125A virus-infected cells revealed some differences from corresponding *wt*ORF1 and V125A cells. Whereas the levels of p85α, p85β, and p110α were higher in *wt*ORF1 cells than in V125A and vector cells (**Figure 2**), these protein levels were higher in both *wt* Ad9 virus- and Ad9-V125 virus-infected cells than in mock-infected cells, with comparable p85α and p110α elevation in the virus-infected cells and somewhat higher p85β elevation in *wt* Ad9 virus-infected cells than in Ad9-V125 virus-infected cells (**Figure 6A**; **Tables S7 and S8**). These results suggested that, in adenovirus-infected cells, p85 and p110 are upregulated by two distinct viral functions, E4-ORF1 and an undetermined viral factor, which differs from E4-ORF1 by inducing weaker elevation of p85β protein levels and not activating PI3K.

### Two carboxyl-terminal residues of E4-ORF1 determine binding to both PI3K and Dlg1

**Figure 7:**
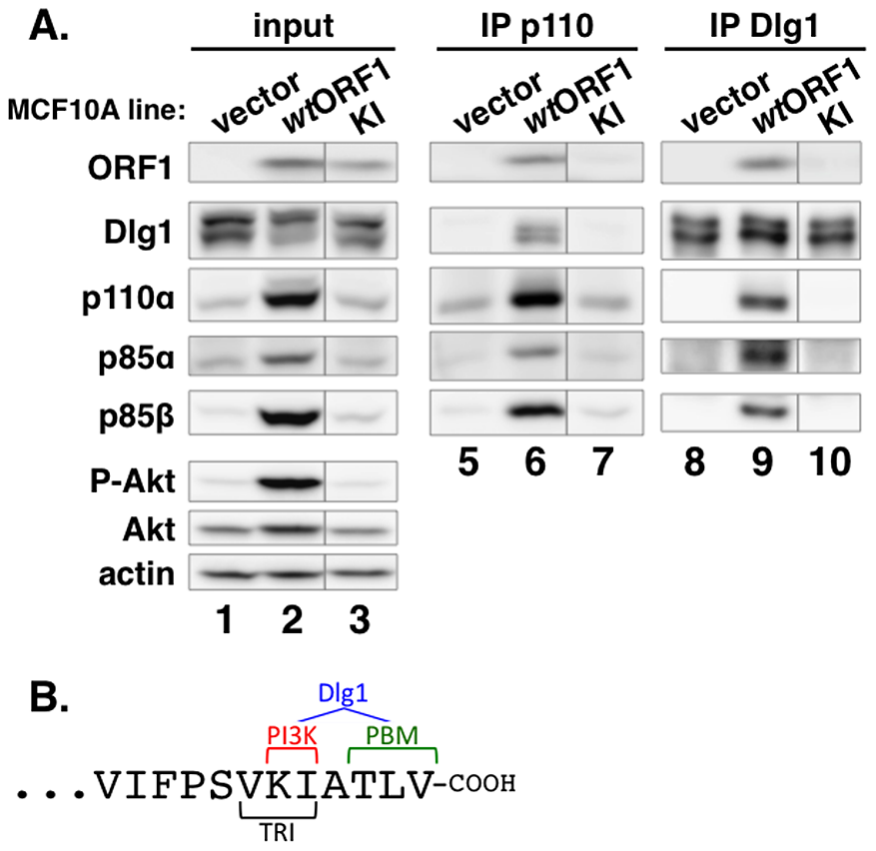
Two adjacent residues located at the E4-ORF1 carboxyl-terminus determine binding to both PI3K and Dlg1. (**A**) E4-ORF1 mutant KI is defective in binding to both PI3K and Dlg1. Co-IP assays were conducted with the indicated MCF10A lines as described in the legend to Figure 5. Vertical lines denote removal of extraneous sample lanes. (**B**) The location of the KI residues at the extreme carboxyl-terminus of E4-ORF1, along with their close proximity to the PBM and overlap with the TRI element, is shown. Also illustrated is that the KI residues determine PI3K binding; both the KI residues and the PBM element, defined by residues TLV, determine Dlg1 binding; and the TRI element, defined by residues VKI, determines E4-ORF1 homo-trimerization.

To identify residues of E4-ORF1 required for binding to PI3K, we generated MCF10A lines expressing 16 different E4-ORF1 mutants. We then IPed p110α from lysates of these cell lines, and tested for co-IP of E4-ORF1 (data not shown). One E4-ORF1 mutant, mutant KI, was defective for binding to PI3K compared to the *wt* E4-ORF1 protein (**Figure 7A**, compare lanes 6 and 7). Mutant KI has two point mutations that change the adjacent carboxyl-terminal residues K120 and I121 to alanine residues (**Figure 7B**) [5]. We also IPed Dlg1 from the lysates and tested for co-IP of E4-ORF1. The data showed that mutant KI is similarly defective for binding to Dlg1 (**Figure 7A**, compare lanes 9 and 10), consistent with a previous report [5]. These binding defects of mutant KI are specific and do not reflect general protein misfolding or complete loss of function because it retains the capacity to form homo-trimers and to bind the cellular PDZ proteins MUPP1, MAGI-1, and ZO-2 [5]. Thus, we identified two E4-ORF1 residues that specifically determine both interactions required to form the ternary complex. Interestingly, the KI residues are located at the extreme carboxyl-terminus near the PBM required for binding to Dlg1, as well as to other PDZ proteins, and additionally overlap the TRI element required for E4-ORF1 homo-trimerization (**Figure 7B**).

### Dlg1 in the ternary complex functions to recruit both E4-ORF1 and PI3K to the plasma membrane

The requirement for Dlg1 to support E4-ORF1-induced PI3K activation correlates with its ability to recruit E4-ORF1 protein to the plasma membrane [34]. This observation, together with discovery of the Dlg1:E4-ORF1:PI3K ternary complex, led us to hypothesize that Dlg1 recruits not only E4-ORF1 but also PI3K to the plasma membrane. This hypothesis predicts that E4-ORF1 and PI3K would co-localize with Dlg1 at the plasma membrane in *wt*ORF1 cells, but not in V125A cells where the PBM mutant V125A protein can neither bind Dlg1 nor activate PI3K, yet can bind PI3K (**Figure 5**). We investigated this idea in indirect immunofluorescence (IF) assays where vector, *wt*ORF1, and V125A cells were double labeled with antibodies to Dlg1 and either E4-ORF1 or p85 and then analyzed by confocal microscopy.

**Figure 8:**
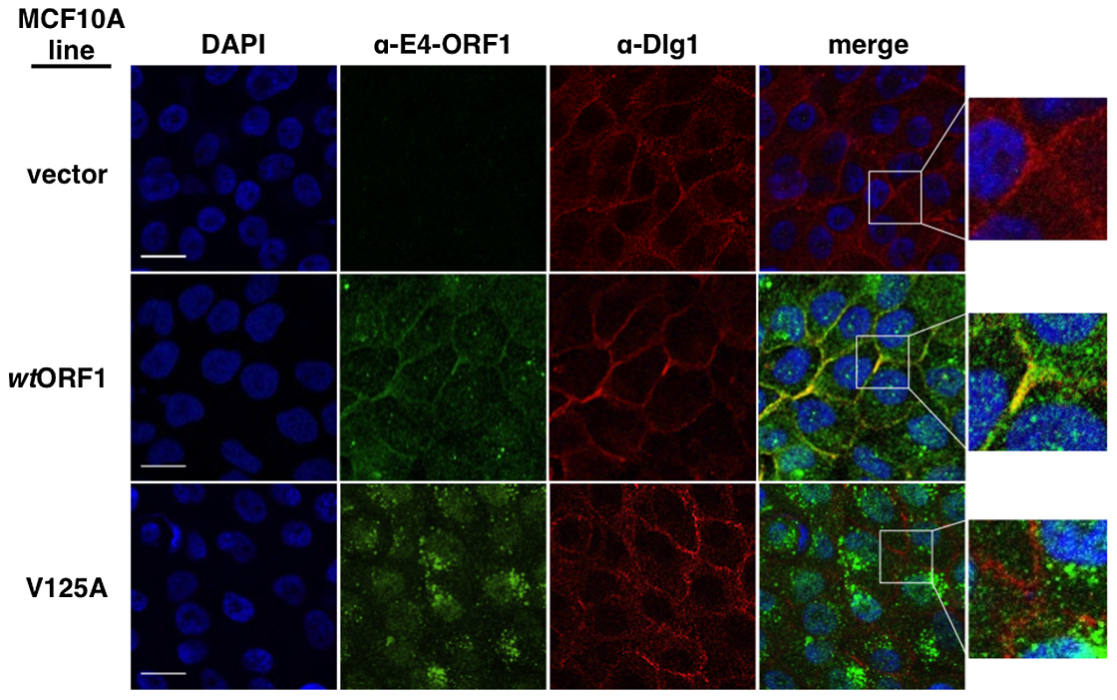
Dlg1 in the ternary complex recruits E4-ORF1 to the plasma membrane. In these indirect immunofluorescence assays, the indicated MCF10A lines were dually stained with E4-ORF1 (green) and Dlg1 (red) antibodies, followed by visualization by fluorescence confocal microscopy, as described in the *Materials and Methods*. Nuclei were counterstained with DAPI (blue). Individual and merged images are shown. White scale bar = 20 µm.

We first examined each cell line for co-localization of E4-ORF1 and Dlg1 (**Figure 8**). In *wt*ORF1 cells, E4-ORF1 protein localized in the cytoplasm, exhibiting both diffuse and punctate distributions, as well as at the plasma membrane. Previous findings indicated that the cytoplasmic punctae reflect a combination of E4-ORF1 monomer sequestration of TJ-associated PDZ proteins and E4-ORF1 trimer association with membrane vesicles whereas the plasma membrane fraction represents E4-ORF1 trimer binding to Dlg1 [5], [34]. Consistent with these findings, the PBM mutant V125A protein failed to localize at the plasma membrane, but retained the cytoplasmic punctate distribution, likely through the PBM-independent association of E4-ORF1 with membrane vesicles [15]. Unlike the *wt* E4-ORF1 protein, the mutant V125A protein also accumulated in the nucleus, a defect of PBM mutants attributed to passive nuclear diffusion resulting from loss of PBM-dependent anchoring to PDZ proteins at extranuclear sites [15]. We also note that in IF assays described here and below, V125A and T123D cells yielded identical results (data not shown). More importantly, while we found that Dlg1 similarly localized at cell-cell contact regions of the plasma membrane in vector, *wt*ORF1, and V125A cells, only the plasma membrane-associated E4-ORF1 protein fraction in *wt*ORF1 cells co-localized with Dlg1. Quantified IF data for this experiment, and other IF experiments detailed below, are presented in **Table S9**.

**Figure 9:**
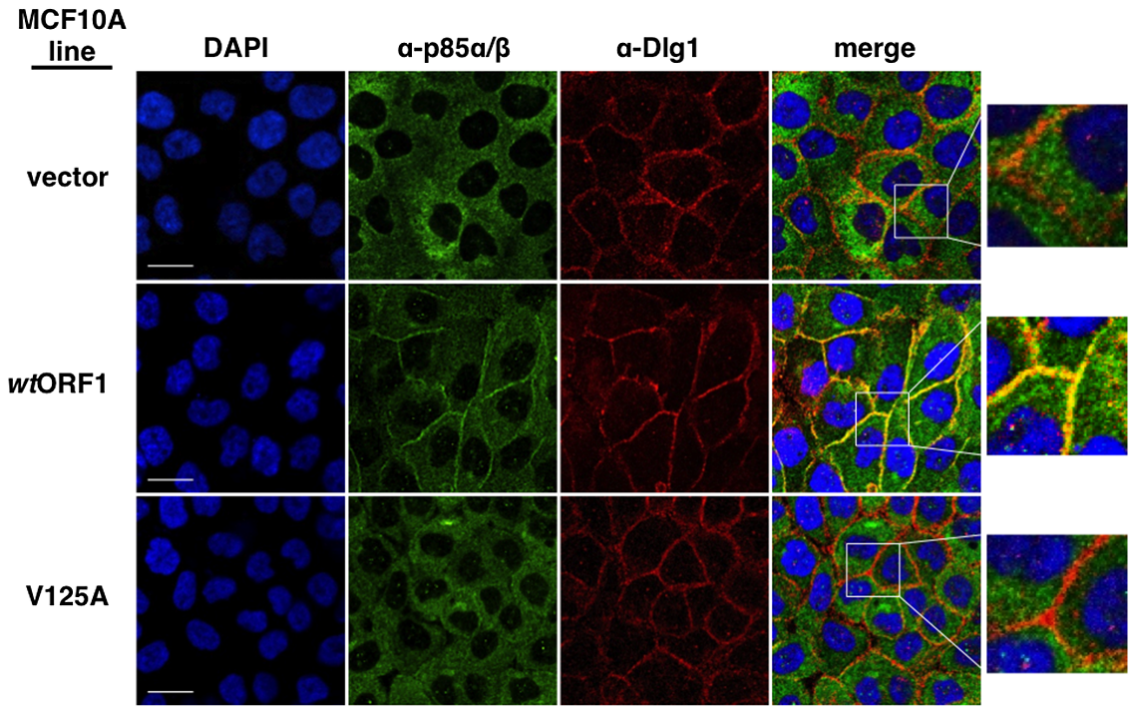
Dlg1 in the ternary complex also recruits PI3K to the plasma membrane. Indirect immunofluorescence assays were performed and analyzed as described in the legend to Figure 8, except the indicated MCF10A lines were dually stained with antibodies to p85α/β (green) and Dlg1 (red). White scale bar = 20 µm.

**Figure 10:**
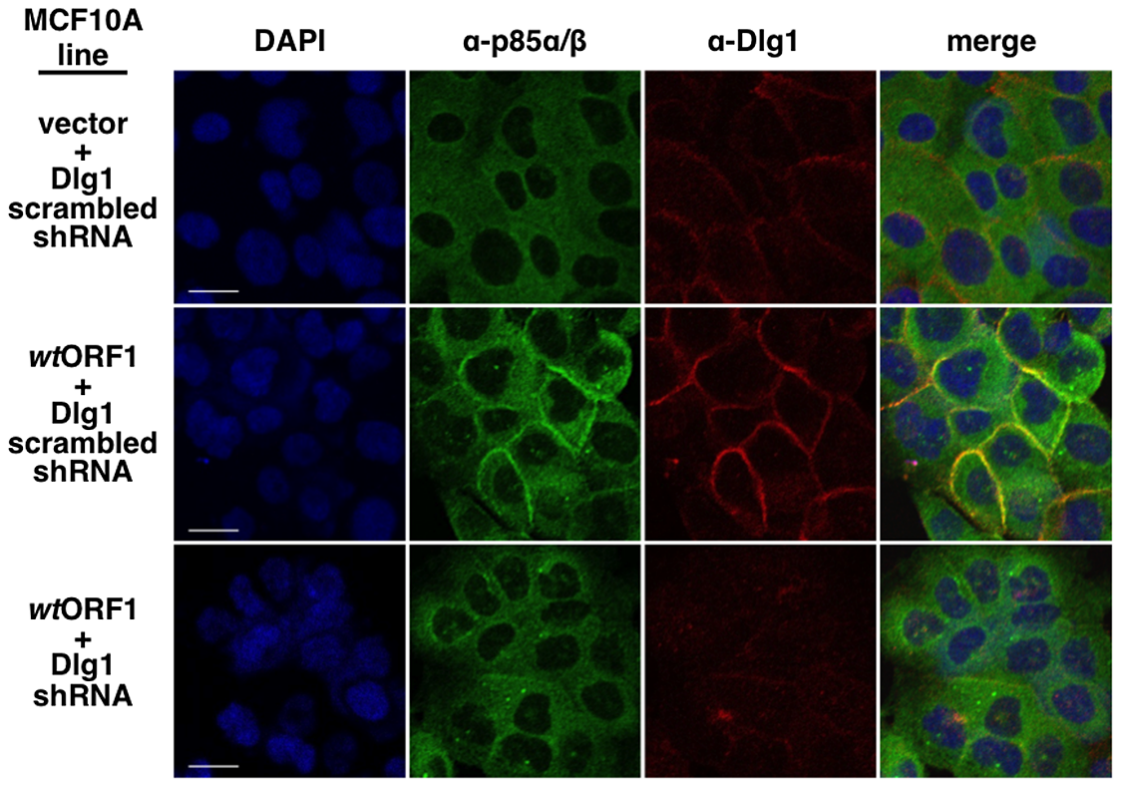
ShRNA-mediated Dlg1 depletion diminishes E4-ORF1-induced recruitment of PI3K to the plasma membrane. Indirect immunofluorescence assays were performed and analyzed as described in the legend to Figure 9 using the indicated MCF10A lines.

We next tested the cell lines for co-localization of p85 and Dlg1 (**Figure 9**). In vector and V125A cells, p85 exhibited a cytoplasmic distribution but, due to an absence at the plasma membrane, it failed to co-localize with Dlg1. In *wt*ORF1 cells, p85 was similarly distributed in the cytoplasm and, more importantly, was additionally detected at the plasma membrane where it co-localized with Dlg1. Furthermore, the latter effect was decreased by shRNA-mediated Dlg1 depletion in *wt*ORF1 cells (**Figure 10**). These data, together with those presented in **Figure 8**, indicated that PBM-mediated binding of the *wt* E4-ORF1 protein to Dlg1 functions not only to assemble the Dlg1:E4-ORF1:PI3K ternary complex but also to localize PI3K to the plasma membrane.

### The ternary complex stimulates PI3K signaling at the plasma membrane

**Figure 11:**
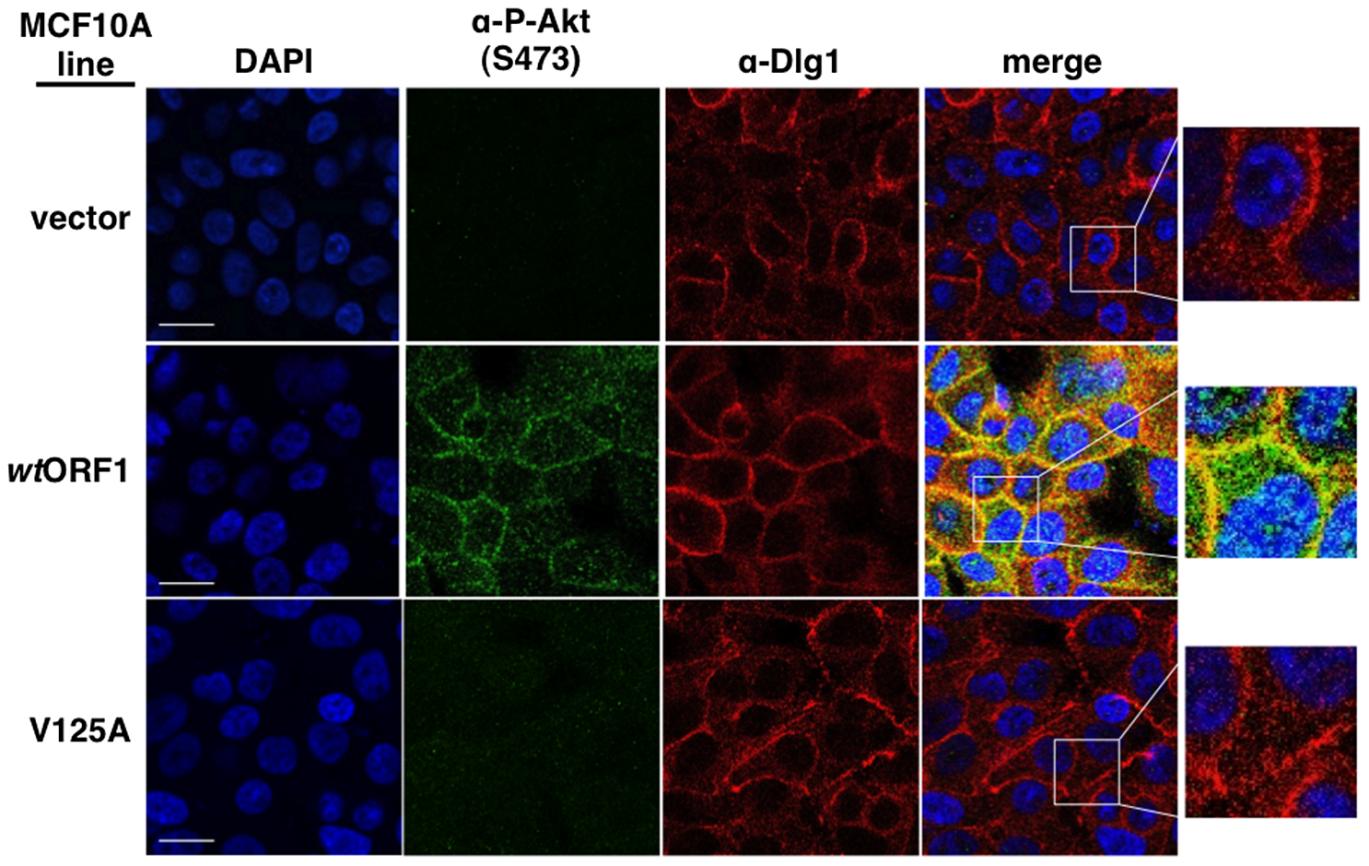
Sites of E4-ORF1-induced activation of PI3K effector Akt co-localize with Dlg1 at the plasma membrane. Indirect immunofluorescence assays were performed and analyzed as described in the legend to Figure 8, except the indicated MCF10A lines were dually stained with antibodies to P-Akt (Ser473) (green) and Dlg1 (red). White scale bar = 20 µm.

**Figure 12:**
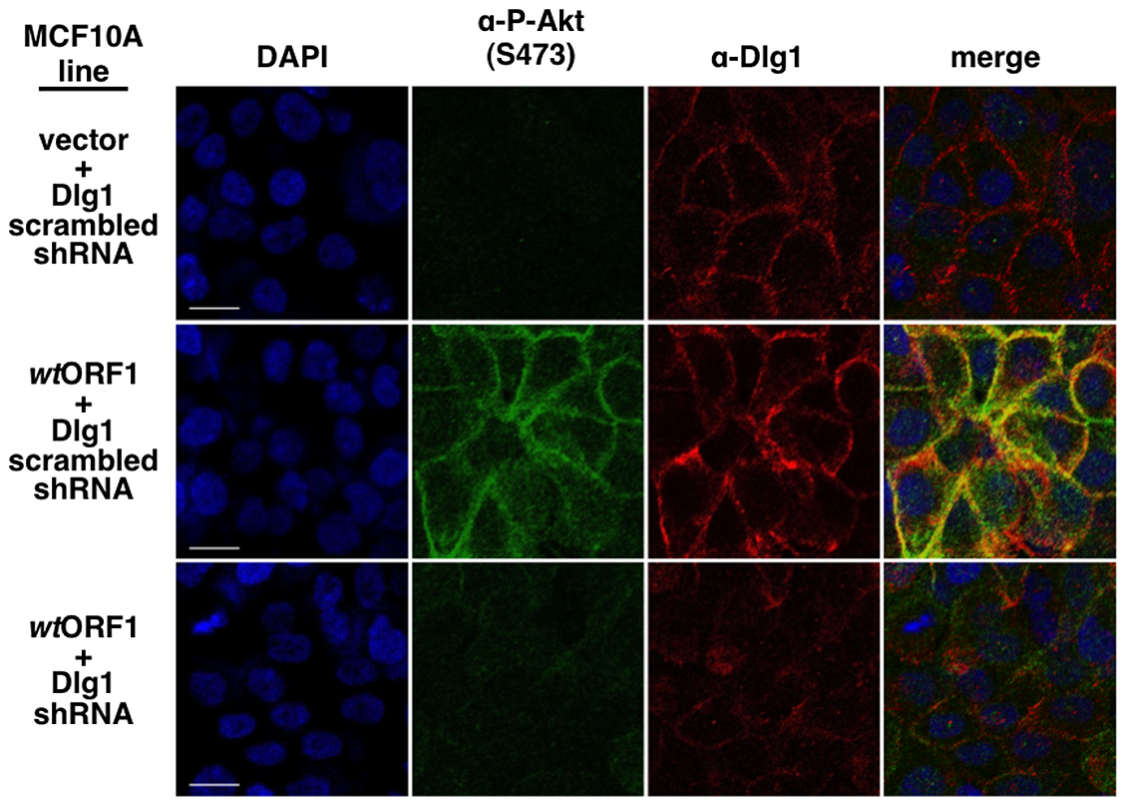
ShRNA-mediated Dlg1 depletion diminishes E4-ORF1-induced recruitment of P-Akt to the plasma membrane. Indirect immunofluorescence assays were performed and analyzed as described in the legend to Figure 11 using the indicated MCF10A lines.

To obtain evidence that the membrane-associated Dlg1:E4-ORF1:PI3K ternary complex mediates PI3K signaling, we investigated whether P-Akt, which is recruited to membrane sites of PI3K activation by binding to PI3K product PIP3, also co-localizes with Dlg1 at the plasma membrane (**Figure 11**). Consistent with results of immunoblot assays shown in **Figures 2** **and** **5**, P-Akt staining was detected in *wt*ORF1 cells but not in vector or V125A cells. In *wt*ORF1 cells, P-Akt distributed in a speckled pattern in the cytoplasm, and also accumulated at the plasma membrane where it co-localized with Dlg1. We observed a similar accumulation of total Akt protein at the plasma membrane of *wt*ORF1 cells but not vector or V125A cells (**Figure S3**). In addition, this accumulation of P-Akt at the plasma membrane was diminished by shRNA-mediated Dlg1 depletion in *wt*ORF1 cells (**Figure 12**). These data supported the conclusion that the ternary complex mediates PI3K signaling at the plasma membrane.

### E4-ORF1 promotes recruitment of cytoplasmic Dlg1 to the plasma membrane

**Figure 13:**
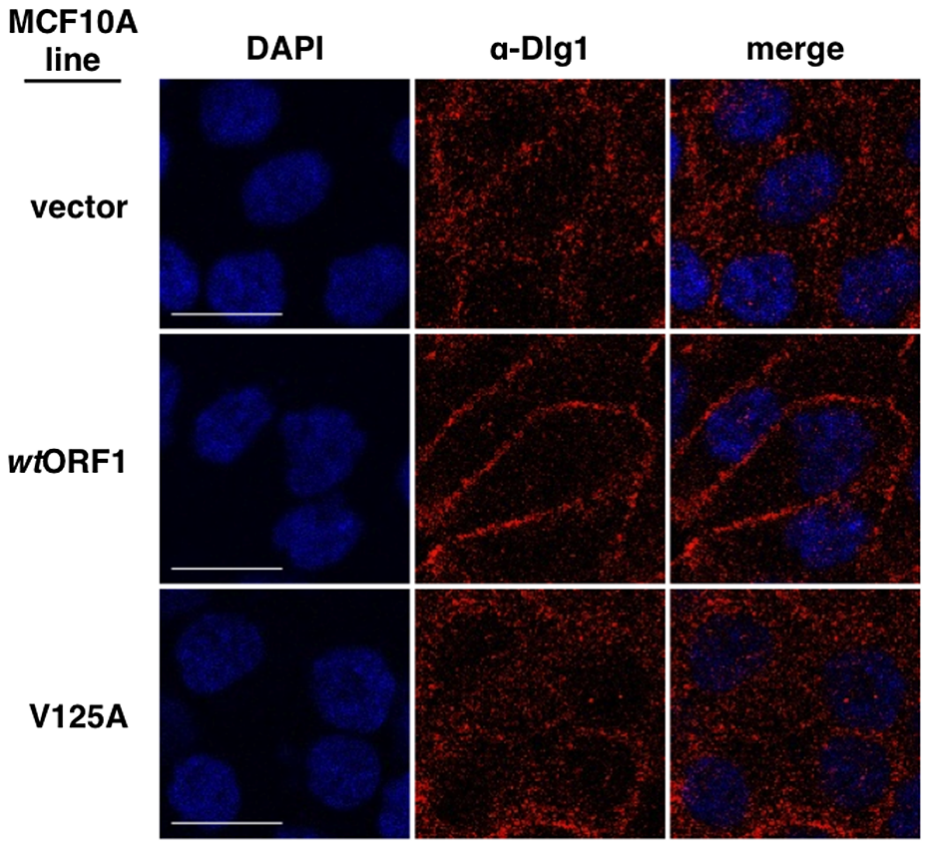
E4-ORF1 promotes translocation of cytoplasmic Dlg1 to the plasma membrane. Indirect immunofluorescence assays were performed and analyzed as described in the legend to Figure 8, except the indicated MCF10A lines were analyzed just after reaching confluency and were stained with antibody to Dlg1 (red). White scale bar = 20 µm.

Results presented in **Figures 8** **and** **9** demonstrated that both E4-ORF1 and PI3K are recruited to Dlg1 located at the plasma membrane of MCF10A cells. We previously reported that E4-ORF1 also induces cytoplasmic Dlg1 to translocate to the plasma membrane [34]. This finding led to the expectation that *wt*ORF1 cells would show higher Dlg1 membrane staining and lower Dlg1 cytoplasmic staining than vector or V125A cells. While this effect was evident in **Figures 10** **and** **12**, it was either weak or absent in **Figures 8** **,** **9** **and** **11**. These variable results can be explained by the fact that prior to formation of adherens junctions, Dlg1 localizes primarily in the cytoplasm whereas during formation of adherens junctions, Dlg1 increasingly concentrates at this site of the plasma membrane, even in the absence of E4-ORF1. In experiments where the E4-ORF1 effect was weak or absent, the cells were post-confluent and had concentrated Dlg1 at mature adherens junctions, thereby partially or completely masking E4-ORF1-induced Dlg1 membrane recruitment. Thus, to optimize detection of this E4-ORF1 activity, we compared the localization of Dlg1 in confluent vector, *wt*ORF1, and V125A cells prior to formation of adherens junctions (**Figure 13**). Under these conditions, *wt*ORF1 cells displayed higher Dlg1 membrane staining and lower cytoplasmic staining than vector or V125A cells. This finding demonstrated that E4-ORF1 also promotes cytoplasmic Dlg1 to translocate to the plasma membrane of MCF10A cells.

### The ternary complex is required for E4-ORF1-induced transformation of human epithelial cells

**Figure 14:**
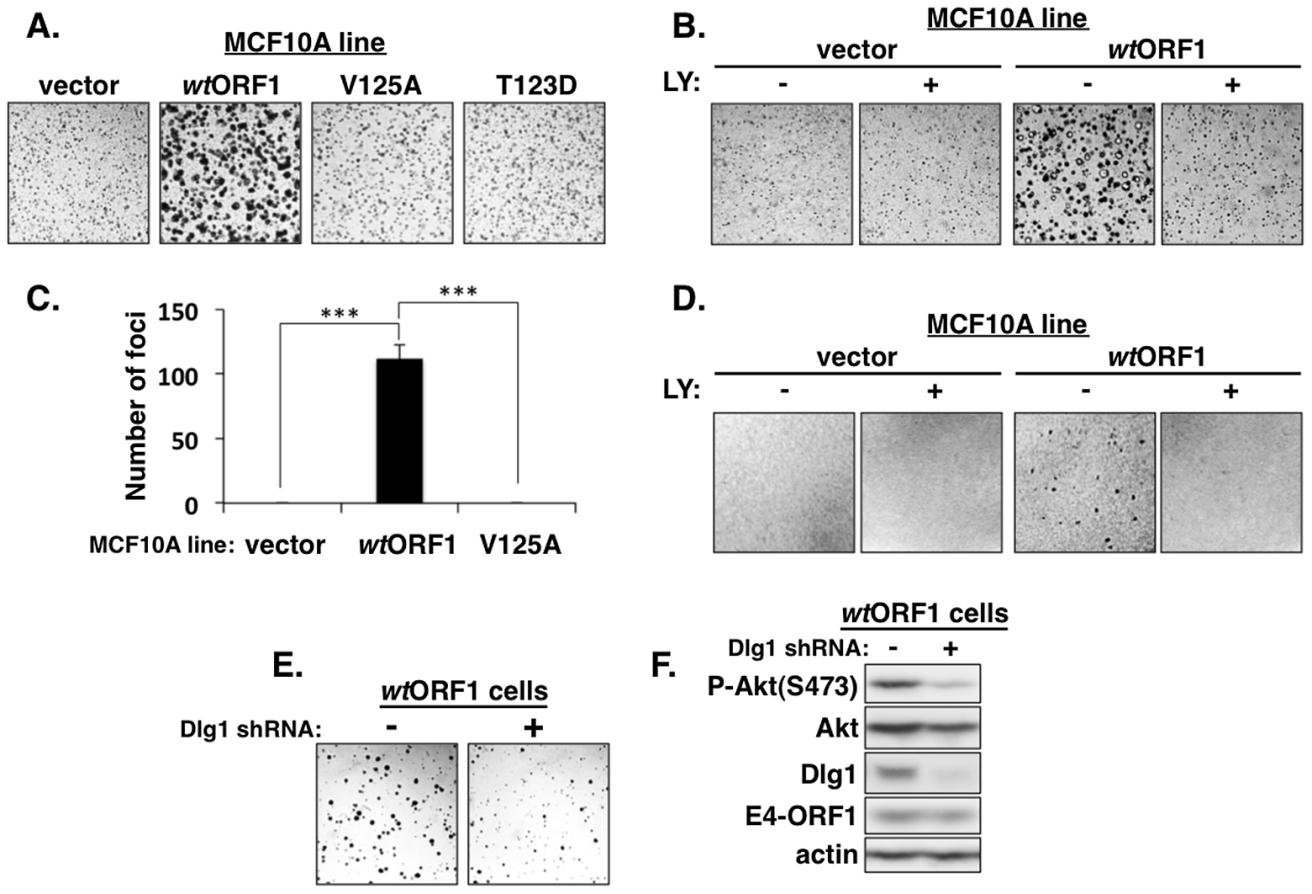
E4-ORF1 induces PBM-, PI3K-, and Dlg1-dependent cellular transformation. (**A**) *Wt*ORF1 cells but not vector, V125A, or T123D cells grow in soft agar. The indicated MCF10A lines were analyzed in soft agar assays as described in the *Materials and Methods*. Cells were photographed 5 days post plating. (40× magnification) (**B**) LY294002 (LY) inhibits soft agar growth of *wt*ORF1 cells. Soft agar assays were performed as in (A), except the culture medium contained either 100 µM LY (+) or DMSO vehicle (−) from 3 days post plating until the experiment was ended at 10 days post plating, when cells were photographed (40× magnification). (**C**) *Wt*ORF1 cells but not vector or V125A cells form foci in monolayer cultures. The indicated MCF10A lines were analyzed in focus formation assays as described in the *Materials and Methods*. Foci were counted at 22 days post plating. The experiment was done in triplicate, and bars indicate the mean ± SD (*p*-value<1.0E-08). (**D**) LY inhibits focus formation by *wt*ORF1 cells. Focus formation assays were performed as described in (C), except a mixture of 150 *wt*ORF1 or vector cells and 1×10^5^ vector cells was seeded into each well. From 6 days post plating until the experiment was ended at 21 days post plating, cells were cultured in complete medium containing 100 µM LY (+) or the same volume of DMSO vehicle (−). Cells were stained and photographed. Panels show a representative 2.4 cm×2.4 cm square region of each cell monolayer. (**E**) Dlg1 depletion decreases the growth of *wt*ORF1 cells in soft agar. Soft agar assays were performed as in (A), except *wt*ORF1 cells stably transduced with pSUPER-Dlg1 shRNA (+) or pSUPER-Dlg1 scrambled control shRNA (−) were analyzed and 10-times fewer cells were plated into culture medium containing a sub-inhibitory dose of LY (25 µM). Cells were photographed at 22 days post plating (40× magnification). (**F**) Control immunoblot assays for panel E compare expression of the indicated proteins in the MCF10A lines treated for 30 min with 25 µM LY.

We next investigated the functional importance of the ternary complex in cells. The capacity of cells to form colonies when suspended in soft agar measures anchorage-independent growth, a property that correlates best with tumorigenic potential [40]. Previous studies showed that Ad9 E4-ORF1-expressing rodent fibroblasts and Ad9-induced mammary tumor cells form colonies in soft agar and that this oncogenic property depends on E4-ORF1-induced PI3K activation [7], [34]. In soft agar assays conducted with the MCF10A lines, we found that *wt*ORF1 cells formed colonies with a high cloning efficiency (95%±1.5% SD; n = 3 independent experiments), whereas V125A and T123D cells behaved like vector cells by not forming colonies (**Figure 14A**). Moreover, the PI3K inhibitor LY abolished colony formation by *wt*ORF1 cells (**Figure 14B**). Transformation assays measuring focus formation by these MCF10A lines yielded similar results (**Figures 14C and 14D**). To implicate Dlg1 directly in E4-ORF1-induced cellular transformation, we compared colony formation by *wt*ORF1 cells expressing the Dlg1 shRNA and control *wt*ORF1 cells expressing the scrambled Dlg1 shRNA (see **Figure 3A**, lanes 3 and 4). Initial experiments, however, failed to detect lower colony formation by the Dlg1 shRNA-expressing *wt*ORF1 cells. Given that, on the average, P-Akt levels in *wt*ORF1 cells are elevated 39- to 63-fold (**Table S2**) and the Dlg1 shRNA reduces these levels by only 3.2- to 3.6-fold (**Table S4**), we considered the possibility that the Dlg1 shRNA-mediated reduction in P-Akt levels may be insufficient to decrease colony formation. To test this idea, we performed the soft agar assays in the presence of the PI3K inhibitor drug LY at the sub-inhibitory dose of 25 µM, which is one-fourth the concentration required to abolish PI3K activation, soft agar growth, and focus formation in MCF10A cells (**Figures 2** **,** **14B, and 14D**). Under these conditions, colony formation by *wt*ORF1 cells expressing the Dlg1 shRNA was substantially reduced compared to control cells (**Figure 14E**), consistent with previous findings [34]. Immunoblot assays confirmed that, when cultured with 25 µM LY, *wt*ORF1 cells expressing the Dlg1 shRNA have lower levels of Dlg1 and P-Akt and but not E4-ORF1 than the control cells (**Figure 14F**). Collectively, our findings supported the conclusion that both the Dlg1:E4-ORF1:PI3K ternary complex and its stimulation of PI3K signaling are essential for the function of E4-ORF1 in cells. The results additionally showed that the *wt* E4-ORF1 protein is capable of promoting transformation of a non-transformed human epithelial cell line.

## Discussion

The human adenovirus E4-ORF1 protein mediates constitutive activation of cellular PI3K, a function shown to promote tumorigenesis in experimental animals and to augment viral replication, prolong survival, and modulate lipid and glucose metabolism in cells [7]–[11]. While it is known that PI3K activation by E4-ORF1 depends on its interaction with the cellular PDZ protein Dlg1 and on localization of the resulting Dlg1:E4-ORF1 complex to the plasma membrane, the underlying molecular mechanism for activation had not been previously determined. We report here the first mechanistic insight into this activity by identifying the PI3K p85:p110 heterodimer as a new cellular target of the E4-ORF1 protein.

**Figure 15:**
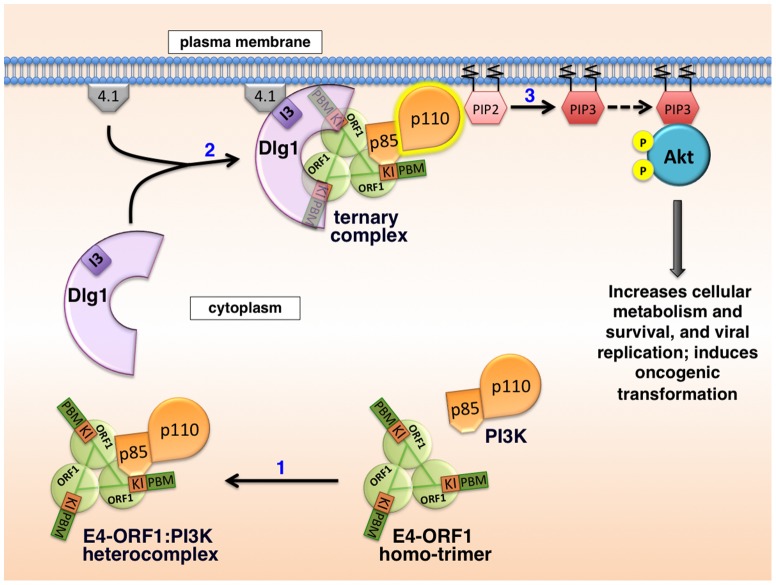
Proposed molecular mechanism for PI3K activation by the adenovirus E4-ORF1 protein. See text of *Discussion* for details.

Based on results reported here along with previously published findings, the mechanism for E4-ORF1-mediated PI3K activation shown in **Figure 15** has emerged. We propose that in step 1, E4-ORF1 via the KI residues binds directly to PI3K to form a cytoplasmic E4-ORF1:PI3K heterocomplex (**Figures 1** **,** **5** **, and** **7**). In step 2, E4-ORF1 within this heterocomplex also interacts with Dlg1-I3 (**Figure 4**) [34], via cooperative binding between two PBM+KI elements in the E4-ORF1 trimer and two PDZ domains in Dlg1 (**Figure 7**) [5], [34], thereby assembling a Dlg1:E4-ORF1:PI3K ternary complex (**Figures 5** **and** **6A**). Our previous finding that only homo-trimeric E4-ORF1 can interact with Dlg1-I3 [5], which is crucial for E4-ORF1-induced PI3K activation (**Figures 3A** **and** **4**) [34], implies that PI3K likewise binds to the E4-ORF1 trimer in both the ternary complex and the E4-ORF1:PI3K heterocomplex, though it remains possible that the E4-ORF1 monomer also binds PI3K. More importantly, a key consequence of assembling the ternary complex is recruitment of the cytoplasmic E4-ORF1:PI3K heterocomplex to the plasma membrane in a Dlg1-dependent fashion (**Figures 8** **–** **10** **and** **13**) [34]. Similar to growth factor receptor- and ras-mediated PI3K activation [12], the ternary complex additionally stimulates PI3K catalytic activity (**Figure 4**) [7]. An interesting possibility is that direct binding of PI3K to both E4-ORF1 and Dlg1 in the ternary complex contributes to the latter effect, as well as to PI3K protein elevation discussed later. In step 3, the increased catalytic activity of PI3K in the ternary complex together with its location at the plasma membrane brings the activated PI3K enzyme into contact with lipid substrate PIP2 to produce PIP3, which in turn recruits Akt to the plasma membrane (**Figure S3**), resulting in its activation by phosphorylation (**Figures 2** **,** **3A** **, and** **11** **–** **12**). The ensuing dysregulated activation of Akt and its downstream effectors enhance viral replication and cellular metabolism and survival, and can also promote cellular transformation (**Figure 14**) [7]–[9], [11], [34]. Also worth mention is that unpublished results suggest that the proposed mechanism of PI3K activation by Ad9 E4-ORF1 protein is shared by E4-ORF1 proteins encoded by other adenovirus serotypes and subgroups (MK and KK, manuscript in preparation).

Numerous viruses encode proteins that dysregulate PI3K in cells [14], [41]–[51], though molecular mechanisms are lacking in many cases. When mechanistic details are available, they often involve direct or indirect viral protein:PI3K interactions mediated by phosphorylated tyrosine residues on the viral protein itself or on an activated cellular receptor bound to the viral protein. Examples include mouse polyomavirus middle T antigen and EBV LMP1 and LMP2A that directly bind PI3K by mimicking tyrosine phosphorylated, activated membrane receptors; HBV X, HIV-1 Nef, and HCV NS5 that directly couple PI3K to non-receptor tyrosine kinases; and HPV E5 that indirectly binds to PI3K through an interaction with activated, cellular receptor protein tyrosine kinases [14]. These examples underscore novel differences in PI3K activation by Ad9 E4-ORF1, including the requirement for Dlg1 (**Figures 3** **and** **4**) and the lack of association of PI3K with tyrosine-phosphorylated proteins [34].

Our data indicated that the ternary complex not only activates PI3K but also elevates p85/p110 protein levels (**Figures 2** **,** **3A** **,** **4** **, and** **7**). The latter effect may be important as PI3K over-expression is reported to dysregulate PI3K signaling. For example, an amplified and over-expressed *PIK3CA* gene coding for the PI3K catalytic subunit hyper-activates PI3K signaling in several different types of human cancers [52], and an over-expressed *wt PIK3CA* cDNA in MCF10A cells increases PI3K signaling and cellular proliferation, and provokes cellular transformation [53]. Because treatment of E4-ORF1-expressing cells with a PI3K inhibitor for 30 min, or 2 h (data not shown), failed to diminish p85/p110 levels yet lowered Akt and ERK levels (**Figure 2**), it seems unlikely that p85/p110 elevation results from the capacity of the downstream PI3K effector complex mTORC1 to increase protein synthesis [52]. We instead postulate that the ternary complex directly mediates Dlg1-dependent p85/p110 protein stability. Results showed that both p85/p110 protein elevation and PI3K activation in *wt*ORF1 cells are diminished by shRNA-mediated Dlg1 depletion or are either severely impaired or absent in V125A and T123D cells (**Figures 2** **and** **3A**), revealing a common dependence on Dlg1. Other data showed that the Dlg1-I2 isoform does not support E4-ORF1-induced PI3K activation but does support E4-ORF1-induced PI3K protein elevation (**Figure 4**), indicating that these two Dlg1-mediated activities are separable. This observation hints to the possible existence of separate pools of ternary complexes containing either Dlg1-I3 or Dlg1-I2. In this scenario, the Dlg1-I2 ternary complex may localize in the cytoplasm and function solely to elevate PI3K protein levels, whereas the Dlg1-I3 ternary complex localizes both in the cytoplasm and at the plasma membrane, with the cytoplasmic complex also functioning to elevate PI3K protein levels and the membrane complex functioning specifically to activate PI3K. Future studies will test these ideas as well as determine whether E4-ORF1-mediated p85/p110 elevation results from increased mRNA levels, protein synthesis, or protein half-life.

Results of soft agar and focus formation assays implicated the ternary complex and its activation of PI3K in E4-ORF1-induced transformation of a human epithelial line that retains features of normal epithelial cells (**Figure 14**). This finding reinforces concerns that the use of adenovirus vectors retaining the *E4-ORF1* gene for vaccination or various therapies, as well as the proposed use of the Ad36 *E4-ORF1* gene to treat fatty liver disease and liver dysfunction or to improve glycemic control [11], may increase patient risk for developing neoplasms.

Evidence also indicates that Dlg1 functions to regulate normal PI3K signaling in cells. For example, Laprise *et al.* showed that Dlg1 is phosphorylated on tyrosine residues at the AJ of human intestinal epithelial cells and that these residues mediate direct binding to and activation of PI3K to promote cellular differentiation [54]. Furthermore, the lipid phosphatase and important human tumor suppressor protein PTEN [13], which antagonizes PI3K signaling by dephosphorylating PIP3, is known to bind Dlg1. This interaction, mediated by the carboxyl-terminal PBM of PTEN and PDZ domain 2 of Dlg1 [55], [56], enhances PTEN activity to block proliferation and viability of MCF-7 breast carcinoma cells and to suppress Schwann cell myelination of peripheral nerves [57], [58]. In addition to human adenovirus, several other pathogenic human viruses code for PBM-containing proteins that target Dlg1 and also bind to and/or activate PI3K, including the E6 oncoprotein of high-risk HPVs [42], [59], [60], the Tax oncoprotein of HTLV-1 [61]–[65], and the NS1 protein of influenza A viruses [66]. These observations suggest that, under normal physiological conditions, Dlg1 functions as a key regulator of PI3K signaling and that pathogenic human viruses commonly hijack this cellular PDZ protein, at least in part, to dysregulate the PI3K pathway and, in doing so, enhance viral infections associated with acute and chronic human diseases and cancer. Hence, studies of human adenovirus and its subversion of cellular PI3K, Dlg1, and other PDZ proteins may yield mechanistic insights that aid development of new therapeutic strategies for treating viral diseases in people.

## Materials and Methods

### Plasmids

Plasmid pBABE-puro or -blasti containing a *wt* or mutant *E4-ORF1* or *rasV12* cDNA, plasmid pGEX-2TK containing a *wt E4-ORF1* cDNA, and plasmid GW1 containing *wt E4-ORF1*, *rasN17*, *HA-ΔNT-Dlg1-I3*, or *HA-ΔNT-Dlg1-I2* cDNA were described [7], [18], [19], [34]. Oligonucleotides encoding Dlg1 shRNA 5′GCAAGATACCCAGAGAGCA3′ or matched scrambled shRNA 5′GGACCACAACGACTAGAGA3′ were cloned into plasmid pSUPER-retro (Oligoengine, Seattle).

### Cells and viruses

Human MCF10A mammary epithelial cells (American Type Culture Collection) were maintained, as described [39], in complete medium consisting of DMEM/F-12 supplemented with 5% horse serum (Invitrogen, Carlsbad, CA), 20 ng/ml epidermal growth factor (EGF) (Peprotech, Rocky Hill, NJ), 100 µg/ml hydrocortisone, 10 µg/ml insulin, 1 ng/ml cholera toxin, and 20 µg/ml gentamicin (Sigma-Aldrich, St. Louis, MO). MCF10A lines were generated by transduction with retroviral vector pBABE and/or pSUPER-retro followed by selection in complete medium containing 2 µg/ml puromycin and/or 25 µg/ml blasticidin. Experiments utilized pools of selected cells passaged five times or less, except for the experiment presented in Figure 12B, which used cells at passage number 8. The same numbers of cells were plated for all experiments comparing different cell lines or treatments. For some experiments, cells were passaged into complete medium containing a lower concentration of EGF (5 ng/ml) [39]. PI3K inhibitor LY294002 was purchased (Cell Signaling Technology, Inc., Beverly, Massachusetts). Transfections were performed with *Trans*IT-LT1 Transfection Reagent (Mirus Bio, Madison, WI). *Wt* and mutant Ad9 viruses and their propagation in human A549 cells were described [7], [16].

### Cell extracts

Extracts were prepared, as described [19], by lysis of cells in ice-cold RIPA buffer (150 mM NaCl, 50 mM Tris-HCl pH 8.0, 1% Nonidet P-40, 0.5% deoxycholate, 0.1% SDS) containing protease inhibitors (2 mM PMSF, 20 µg/ml each of leupeptin and aprotinin) and phosphatase inhibitors (50 mM NaF, 10 mM sodium pyrophosphate, 1 mM sodium orthovanadate). Protein concentrations were determined by the Bradford method.

For cell fractionation assays [15], cells were lysed in RIPA buffer and then centrifuged (10,000× *g* for 15 min at 4°C) to separate the detergent-soluble supernatant and detergent-insoluble pellet fractions. The pellet fraction was subsequently solubilized in a volume of 2× sample buffer (125 mM Tris-HCl, pH 6.8, 4% SDS, 0.2 M DTT, 20% glycerol, 0.001% bromophenol blue) equal to that of the detergent-soluble supernatant fraction. Experiments compared equal volumes of the detergent-soluble supernatant and detergent-insoluble pellet fractions.

### Antibodies and purified recombinant human PI3K

Ad9 E4-ORF1 antiserum was described [3]. Antibodies to p110α, Akt, phospho-Akt(Ser473), phospho-Akt(Thr308), p42/44 MAPK and phospho-p42/44 MAPK (Thr202/Tyr204) (Cell Signaling Technologies), or p85β, SAP97 (Dlg1), and scribble (Santa Cruz Biotechnologies), or p85α, p85α/β, and actin (Millipore), or ras (BD Biosciences), or HA (Sigma-Aldrich) were purchased. Greater than 90% pure, catalytically active recombinant PI3K generated by co-expression of histidine-tagged full-length human p85α and p110α in Sf9 cells was purchased (SignalChem).

### Pulldowns, immunoprecipitations, immunoblots, and mass spectrometry

GST pulldowns and immunoprecipitations with glutathione-sepharose beads or protein G-sepharose beads (GE Healthcare Life Sciences), respectively, were carried out as described [4], [18]. Recovered proteins and cell extract (30 µg of protein) were resolved by SDS-PAGE, transferred to a PVDF membrane, and immunoblotted as described [4], [18]. Immunoblotted membranes were imaged with a UVP Biospectrum 810 Imaging System (Upland, CA) and analyzed with VisionWorksLS software. Differences in the levels of specified proteins between two cell samples were quantified by comparing protein band intensities normalized to actin, and the Student's t-test was performed to determine statistical significance. E4-ORF1-binding proteins were identified by conducting a pulldown assay with extracts of suspension-cultured HeLa cells, resolving recovered proteins by SDS-PAGE, digesting separate gel sections with trypsin, and subjecting the released peptides to MALDI-TOF mass spectrometry.

### Indirect immunofluorescence and confocal microscopy

Glass slides (Millicell EZ SLIDE, Millipore) were coated with poly-L-lysine (Sigma-Aldrich). Cells plated on the slides were fixed in 2% formaldehyde (Polysciences, Inc.), permeabilized with 0.5% Triton X-100, quenched with 100 mM glycine, blocked in 10% goat serum, and incubated with primary antibody and then with Alexa Fluor 488-conjugated goat anti-rabbit IgG and/or Alexa Fluor 594-conjugated goat anti-mouse IgG secondary antibodies (Life Technologies Corp.). Prior or following incubation with primary antibody, cells were washed between each step with either phosphate-buffered saline (PBS) or immunofluorescence buffer (IFB) [7.7 mM sodium azide, 0.1% (w/v) BSA, 0.2% (v/v) Triton X-100, 0.05% (v/v) Tween-20 in PBS], respectively. Coverslips were mounted on slides with SlowFade Gold antifade reagent (Life Technologies Corp.). Cells were analyzed by confocal microscopy with a Nikon A1-Rs inverted laser-scanning microscope and NIS Elements software. The percentage of cells in which specified proteins localized at the plasma membrane was quantified using Image J software (NIH).

### Soft agar and focus formation assays

Soft agar assays were carried out as described [3]. Briefly, in complete medium, 3×10^5^ cells were suspended in 1 ml of 0.4% noble agar (Affymetrix) and placed atop a 2 ml 0.8% noble agar underlay in a 6-well plate. Cells were fed complete medium every other day. Colonies were documented with a Nikon D70S camera mounted on a Nikon TMS inverted microscope. ImageJ software (NIH) was used to score the numbers of cells that (a) did and (b) did not form a colony, and cloning efficiency (a/a+b) was calculated from >300 scored cells per experiment. For focus formation assays, 300 *wt*ORF1, V125A, or vector cells mixed with 3×10^5^ vector cells in complete medium were seeded into a 6-well plate. Cells were fed every 3 days and, when visible, foci were stained with crystal violet (5 mg/ml in 25% methanol) and photographed with a Canon PowerShot A1200 digital camera. ANOVA with Tukey post-hoc analysis was performed to determine statistical significance.

### Statistical analyses

Microsoft Excel was used to calculate the mean, standard deviation (SD), and standard error of the mean (SEM) for data. R statistical software was used to determine statistical significance. Standard denotation of asterisks for p values was used (*, p<0.05; **, p<0.01; ***, p<0.001).

### Accession numbers

UniProtKB/Swiss-Prot accession numbers (parentheses) are indicated for proteins mentioned in text: Abl1 (P00519), actin (P68032), Ad9 E4-ORF1 (P89079), Ad9 hexon (Q9QPU1), Akt (P31749), DDX17 (Q92841), Dlg1 (Q12959), dUTPase (P33316), EBV LMP1 (P03230), EBV LMP2A (P13285), ELAV1 (Q15717), ERK1 (P27361), ERK2 (P28482), Ras (P01112), HBV X (Q69027), HCV NS5 (C1IEN6), HIV-1 Nef (P04601), HPV-16 E5 (P06927), HPV-16 E6 (P03126), HTLV-1 Tax (P03409), IGF2BP1 (Q9NZI8), IGF2BP3 (O00425)

Influenza virus A NS1 (P03496), LARP1 (Q6PKG0), LARP2 (Q659C4), MAGI-1 (Q96QZ7), MUPP1 (O75970), p110α (P42336), p85α (P27986), p85β (O00459), PATJ (Q8NI35), PDK1 (Q15118), PTEN (P60484), scribble (Q14160), UPF2 (Q9HAU5), ZO-2 (Q9UDY2)

## Supporting Information

Figure S1 **The vast majority of E4-ORF1 protein is contained within the insoluble pellet fraction of MCF10A cells.** Soluble (S) and insoluble (I) extract fractions of vector and *wt*ORF1 cells were prepared (see *Materials and Methods*). An equivalent amount of each fraction was analyzed in an immunoblot assay with E4-ORF1 antibody. The soluble fraction was additionally immunoblotted with actin antibody as a loading control.
(TIFF)

Figure S2 **Dominant-negative mutant rasN17 blocks PI3K activation by mutant rasV12 but not E4-ORF1.** Mutant rasN17 (**A**) blocks mutant rasV12-induced PI3K activation but (**B**) has no effect on E4-ORF1-induced PI3K activation. MCF10A cells were transfected with expression plasmid GW1-rasN17 (2 µg), GW1-E4-ORF1 (75 ng), or GW1-rasV12 (500 ng) alone or in the indicated combinations. The total amount of DNA in each transfection was equalized to 7.575 µg using empty GW1 plasmid. At 48 h post-transfection, cells were serum starved for 1 h, and then extracts were prepared and analyzed in immunoblot assays using antibodies to the indicated proteins. The insoluble (I) pellet fraction (see *Materials and Methods*) was immunoblotted for E4-ORF1 protein. Vertical lines in (A) denote removal of extraneous sample lanes.
(TIFF)

Figure S3 **Total Akt accumulates at the plasma membrane in** ***wt*** **ORF1 cells.** In IF assays, the indicated MCF10A lines stained with an antibody reactive to total Akt (green) were visualized by fluorescence confocal microscopy. Nuclei were counterstained with DAPI (blue). Individual and merged images are shown. White scale bar = 20 µm.
(TIFF)

Table S1 **Cellular proteins identified to interact with GST-E4-ORF1 in a pulldown assay.** Shown are 10 selected proteins identified by mass spectrometry to bind the Ad9 E4-ORF1 fusion protein in a pulldown assay conducted with extracts from HeLa cells.
(DOCX)

Table S2 **Average fold changes in protein levels quantified from immunoblots of** ***wt*** **ORF1** ***versus*** **vector cells.** For Figures 2 and 3A, average fold changes in levels of the indicated proteins were quantified from independent immunoblots of *wt*ORF1 cells *versus* vector cells. See *Materials and Methods* for details.
(DOCX)

Table S3 **Average fold changes in protein levels quantified from immunoblots of rasV12** ***versus*** **vector cells.** For Figures 2 and 3B, average fold changes in levels of the indicated proteins were quantified from independent immunoblots of *rasV12* cells *versus* vector cells. See *Materials and Methods* for details.
(DOCX)

Table S4 **Average fold reductions in protein levels quantified from immunoblots of** ***wt*** **ORF1 cells transduced** ***versus*** **not transduced with the Dlg1 shRNA vector.** For Figure 3A, average fold reductions in levels of the indicated proteins were quantified from independent immunoblots of *wt*ORF1 cells transduced with the Dlg1 shRNA vector *versus* the matched scrambled shRNA vector. See *Materials and Methods* for details.
(DOCX)

Table S5 **Average fold increases in protein levels quantified from immunoblots of Dlg1 shRNA-expressing MCF10A cells transfected with E4-ORF1 plasmid in combination with HA-ΔNT-Dlg1-I3 plasmid** ***versus*** **E4-ORF1 plasmid alone.** For Figure 4, average fold changes in levels of the indicated proteins were quantified from independent immunoblots of the Dlg1 shRNA-expressing MCF10A line transfected with E4-ORF1 plasmid in combination with HA-ΔNT-Dlg1-I3 plasmid *versus* E4-ORF1 plasmid alone. See *Materials and Methods* for details.
(DOCX)

Table S6 **Average fold changes in protein levels quantified from immunoblots of Dlg1 shRNA-expressing MCF10A cells transfected with E4-ORF1 plasmid in combination with HA-ΔNT-Dlg1-I2 plasmid** ***versus*** **E4-ORF1 plasmid alone.** For Figure 4, average fold changes in levels of the indicated proteins were quantified from independent immunoblots of the Dlg1 shRNA-expressing MCF10A line transfected with E4-ORF1 plasmid in combination with HA-ΔNT-Dlg1-I2 plasmid *versus* E4-ORF1 plasmid alone. See *Materials and Methods* for details.
(DOCX)

Table S7 **Average fold changes in protein levels quantified from immunoblots of Ad9 virus-** ***versus*** **mock-infected cells.** For Figure 6A, average fold changes in levels of the indicated proteins were quantified from independent immunoblots of *wt* Ad9 virus-infected cells *versus* mock-infected cells. See *Materials and Methods* for details.
(DOCX)

Table S8 **Average fold changes in protein levels quantified from immunoblots of Ad9-V125A virus-** ***versus*** **mock-infected cells.** For Figure 6A, average fold changes in levels of the indicated proteins were quantified from independent immunoblots of Ad9-V125A virus-infected cells *versus* mock-infected cells. See *Materials and Methods* for details.
(DOCX)

Table S9 **Average percentage of vector cells** ***versus wt*** **ORF1 cells showing E4-ORF1, p85, P-Akt, and Akt protein staining at the plasma membrane in IF assays.** For Figures 8, 9, 11, and S3, the average percentages of vector cells *versus wt*ORF1 cells exhibiting plasma membrane staining for the indicated proteins were quantified from independent IF assays. See *Materials and Methods* for details.
(DOCX)
