# Supplementary figures and images for: The Human Adenovirus E4-ORF1 Protein Subverts Discs Large 1 to Mediate Membrane Recruitment and Dysregulation of Phosphatidylinositol 3-Kinase
Source: PLoS Pathog. 2014 May 1;10(5):e1004102. doi: 10.1371/journal.ppat.1004102 (PMC4006922; doi:10.1371/journal.ppat.1004102)

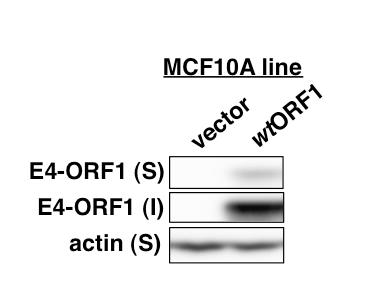

Supplement: Figure S1 — The vast majority of E4-ORF1 protein is contained within the insoluble pellet fraction of MCF10A cells. Soluble (S) and insoluble (I) extract fractions of vector and wtORF1 cells were prepared (see Materials and Methods ). An equivalent amount of each fraction was analyzed in an immunoblot assay with E4-ORF1 antibody. The soluble fraction was additionally immunoblotted with actin antibody as a loading control. (TIFF) [file ppat.1004102.s001.tiff]

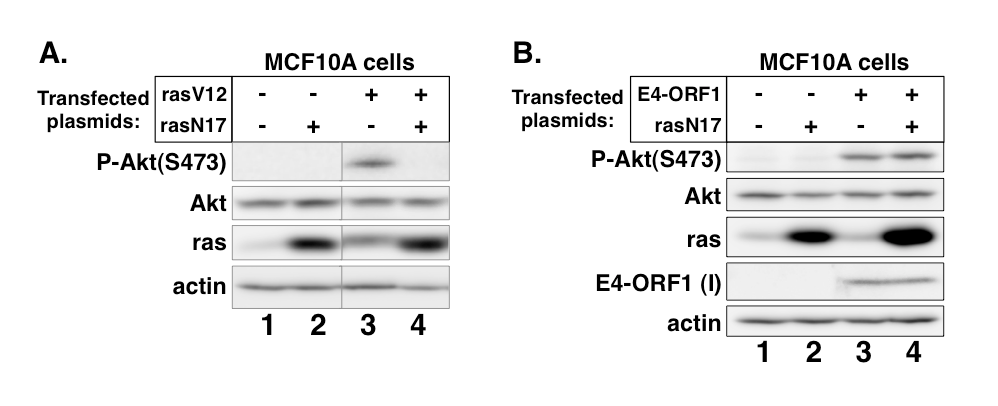

Supplement: Figure S2 — Dominant-negative mutant rasN17 blocks PI3K activation by mutant rasV12 but not E4-ORF1. Mutant rasN17 (A) blocks mutant rasV12-induced PI3K activation but (B) has no effect on E4-ORF1-induced PI3K activation. MCF10A cells were transfected with expression plasmid GW1-rasN17 (2 µg), GW1-E4-ORF1 (75 ng), or GW1-rasV12 (500 ng) alone or in the indicated combinations. The total amount of DNA in each transfection was equalized to 7.575 µg using empty GW1 plasmid. At 48 h post-transfection, cells were serum starved for 1 h, and then extracts were prepared and analyzed in immunoblot assays using antibodies to the indicated proteins. The insoluble (I) pellet fraction (see Materials and Methods ) was immunoblotted for E4-ORF1 protein. Vertical lines in (A) denote removal of extraneous sample lanes. (TIFF) [file ppat.1004102.s002.tiff]

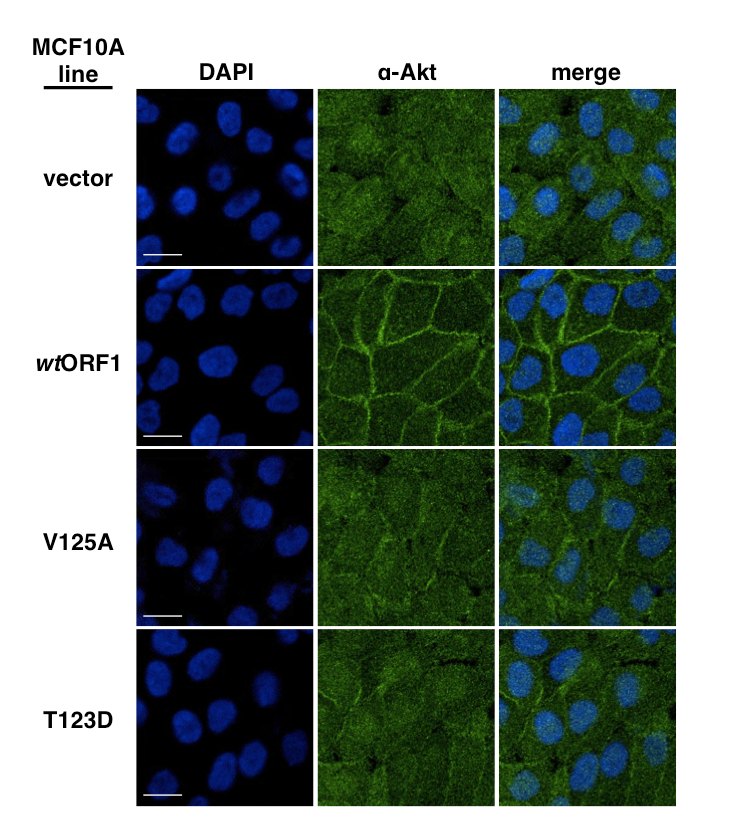

Supplement: Figure S3 — Total Akt accumulates at the plasma membrane in wt ORF1 cells. In IF assays, the indicated MCF10A lines stained with an antibody reactive to total Akt (green) were visualized by fluorescence confocal microscopy. Nuclei were counterstained with DAPI (blue). Individual and merged images are shown. White scale bar = 20 µm. (TIFF) [file ppat.1004102.s003.tiff]
