# Supplementary material for: The Human Adenovirus E4-ORF1 Protein Subverts Discs Large 1 to Mediate Membrane Recruitment and Dysregulation of Phosphatidylinositol 3-Kinase
Source: PLoS Pathog. 2014 May 1;10(5):e1004102. doi: 10.1371/journal.ppat.1004102 (PMC4006922; doi:10.1371/journal.ppat.1004102)
Supplement: Table S1 — Cellular proteins identified to interact with GST-E4-ORF1 in a pulldown assay. Shown are 10 selected proteins identified by mass spectrometry to bind the Ad9 E4-ORF1 fusion protein in a pulldown assay conducted with extracts from HeLa cells. (DOCX) [file ppat.1004102.s004.docx]

| **Table S1.** Selected proteins identified by mass spectrometry to associate with the GST‑Ad9 E4‑ORF1 fusion protein in a pulldown assay | | | |
| --- | --- | --- | --- |
| **GST-Ad9 E4-ORF1-Binding Proteins** | **Entrez**  **Gene ID #** | **Symbol** | **No. of peptides** |
| Phosphoinositide-3-kinase, regulatory subunit 2 beta | 5296 | PIK3R2 | 16 |
| Abl-interactor 1 | 10006 | ABI1 | 13 |
| UPF2 regulator of nonsense transcripts homolog (UPF1) | 26019 | UPF2 | 5 |
| Phosphoinositide-3-kinase, regulatory subunit 1 alpha | 5295 | PIK3R1 | 3 |
| La ribonucleoprotein domain family, member 1 (LARP4) | 23367 | LARP1 | 3 |
| Embryonic lethal, abnormal vision, Drosophila-like 1 | 1994 | ELAVL1 | 3 |
| Insulin-like growth factor 2 mRNA binding protein 1 | 10642 | IGF2BP1 | 2 |
| Insulin-like growth factor 2 mRNA binding protein 3 | 10643 | IGF2BP3 | 1 |
| La ribonucleoprotein domain family, member 2 (LARP4) | 55132 | LARP2 | 1 |
| DEAD box polypeptide 17 (DDX6) | 10521 | DDX17 | 1 |

Yellow: Novel E4-ORF1-binding proteins investigated in this study.

Blue: Also identified as Ad5 E4-ORF1-binding proteins by Rozenblatt-Rosen *et al.* [[38](#_ENREF_38)].

Green: Related to proteins in parentheses also identified as Ad5 E4-ORF1-binding proteins by Rozenblatt-Rosen *et al*. [[38](#_ENREF_38)].
