# Supplementary material for: The Human Adenovirus E4-ORF1 Protein Subverts Discs Large 1 to Mediate Membrane Recruitment and Dysregulation of Phosphatidylinositol 3-Kinase
Source: PLoS Pathog. 2014 May 1;10(5):e1004102. doi: 10.1371/journal.ppat.1004102 (PMC4006922; doi:10.1371/journal.ppat.1004102)
Supplement: Table S2 — Average fold changes in protein levels quantified from immunoblots of wt ORF1 versus vector cells. For Figures 2 and 3A, average fold changes in levels of the indicated proteins were quantified from independent immunoblots of wtORF1 cells versus vector cells. See Materials and Methods for details. (DOCX) [file ppat.1004102.s005.docx]

| **Table S2.** Average fold changes in protein levels quantified from immunoblots of *wt*ORF1 *versus* vector cells | | | | |
| --- | --- | --- | --- | --- |
| **Protein** | **Average fold change** | **SD or**  **(SEM)** | **No. of experiments** | ***p*-value** |
| p110α | +16 | 9.3 | 5 | 6.9E-03** |
| p85α | +16 | 12 | 5 | 2.0E-02* |
| p85β | +40 | 21 | 4 | 1.1E-02* |
| P-Akt(S473) | +39 | 8.6 | 4 | 1.2E-04*** |
| P-Akt(T308) | +63 | (14) | 2 | N/A |
| Akt | +4.7 | 2.5 | 5 | 1.2E-02* |
| Dlg1 | -1.9 | (0.07) | 2 | N/A |
