# Supplementary material for: The Human Adenovirus E4-ORF1 Protein Subverts Discs Large 1 to Mediate Membrane Recruitment and Dysregulation of Phosphatidylinositol 3-Kinase
Source: PLoS Pathog. 2014 May 1;10(5):e1004102. doi: 10.1371/journal.ppat.1004102 (PMC4006922; doi:10.1371/journal.ppat.1004102)
Supplement: Table S3 — Average fold changes in protein levels quantified from immunoblots of rasV12 versus vector cells. For Figures 2 and 3B, average fold changes in levels of the indicated proteins were quantified from independent immunoblots of rasV12 cells versus vector cells. See Materials and Methods for details. (DOCX) [file ppat.1004102.s006.docx]

| **Table S3.** Average fold changes in protein levels quantified from immunoblots of rasV12 *versus* vector cells | | | | |
| --- | --- | --- | --- | --- |
| **Protein** | **Average fold change** | **SD or**  **(SEM)** | **No. of experiments** | ***p*-value** |
| p110α | +25 | 14 | 5 | 1.5E-02* |
| p85α | +26 | 16 | 5 | 2.0E-02* |
| p85β | +18 | 8.9 | 4 | 9.4E-03** |
| P-Akt(S473) | +29 | 15 | 4 | 3.1E-02* |
| P-Akt(T308) | +82 | N/A | 1 | N/A |
| Akt | +11 | 7.3 | 5 | 3.4E-02* |
| Dlg1 | -5.5 | (0.12) | 2 | N/A |
| P-ERK1/2 | +38 | (14) | 2 | N/A |
| ERK1/2 | +53 | (16) | 1 | N/A |
