# Supplementary material for: The Human Adenovirus E4-ORF1 Protein Subverts Discs Large 1 to Mediate Membrane Recruitment and Dysregulation of Phosphatidylinositol 3-Kinase
Source: PLoS Pathog. 2014 May 1;10(5):e1004102. doi: 10.1371/journal.ppat.1004102 (PMC4006922; doi:10.1371/journal.ppat.1004102)
Supplement: Table S4 — Average fold reductions in protein levels quantified from immunoblots of wt ORF1 cells transduced versus not transduced with the Dlg1 shRNA vector. For Figure 3A, average fold reductions in levels of the indicated proteins were quantified from independent immunoblots of wtORF1 cells transduced with the Dlg1 shRNA vector versus the matched scrambled shRNA vector. See Materials and Methods for details. (DOCX) [file ppat.1004102.s007.docx]

| **Table S4.** Average fold reductions in protein levels quantified from immunoblots of *wt*ORF1 cells transduced with the Dlg1 shRNA vector *versus* the negative-control scrambled shRNA vector | | | |
| --- | --- | --- | --- |
| **Protein** | **Average fold reduction** | **SEM** | **No. of experiments** |
| p110α | -2.2 | 0.15 | 2 |
| p85α | -2.7 | 0.69 | 2 |
| p85β | -2.4 | 0.18 | 2 |
| P-Akt(S473) | -3.2 | 0.19 | 2 |
| P-Akt(T308) | -3.6 | 0.37 | 2 |
| Akt | -1.8 | 0.09 | 2 |
| Dlg1 | -3.1 | 0.04 | 2 |
