# Supplementary material for: The Human Adenovirus E4-ORF1 Protein Subverts Discs Large 1 to Mediate Membrane Recruitment and Dysregulation of Phosphatidylinositol 3-Kinase
Source: PLoS Pathog. 2014 May 1;10(5):e1004102. doi: 10.1371/journal.ppat.1004102 (PMC4006922; doi:10.1371/journal.ppat.1004102)
Supplement: Table S5 — Average fold increases in protein levels quantified from immunoblots of Dlg1 shRNA-expressing MCF10A cells transfected with E4-ORF1 plasmid in combination with HA-ΔNT-Dlg1-I3 plasmid versus E4-ORF1 plasmid alone. For Figure 4, average fold changes in levels of the indicated proteins were quantified from independent immunoblots of the Dlg1 shRNA-expressing MCF10A line transfected with E4-ORF1 plasmid in combination with HA-ΔNT-Dlg1-I3 plasmid versus E4-ORF1 plasmid alone. See Materials and Methods for details. (DOCX) [file ppat.1004102.s008.docx]

| **Table S5.** Average fold increases in protein levels quantified from immunoblots of Dlg1 shRNA-expressing MCF10A cells transfected with 75 ng of E4-ORF1 plasmid in combination with an amount of HA‑ΔNT‑Dlg1-I3 plasmid ranging from 3 to 7.5 μg *versus* 75 ng of E4-ORF1 plasmid alone | | | | |
| --- | --- | --- | --- | --- |
| **Protein** | **Average fold change** | **SD** | **No. of experiments** | ***p*-value** |
| P-Akt(S473) | +2.4 | 0.73 | 10 | 2.5E-04*** |
| p110α | +2.3 | 0.93 | 10 | 1.9E-03** |
| p85α/β | +1.9 | 0.48 | 6 | 6.3E-03** |
