# Supplementary material for: The Human Adenovirus E4-ORF1 Protein Subverts Discs Large 1 to Mediate Membrane Recruitment and Dysregulation of Phosphatidylinositol 3-Kinase
Source: PLoS Pathog. 2014 May 1;10(5):e1004102. doi: 10.1371/journal.ppat.1004102 (PMC4006922; doi:10.1371/journal.ppat.1004102)
Supplement: Table S7 — Average fold changes in protein levels quantified from immunoblots of Ad9 virus- versus mock-infected cells. For Figure 6A, average fold changes in levels of the indicated proteins were quantified from independent immunoblots of wt Ad9 virus-infected cells versus mock-infected cells. See Materials and Methods for details. (DOCX) [file ppat.1004102.s010.docx]

| **Table S7.** Average fold changes in protein levels quantified from immunoblots of Ad9 virus- *versus* mock-infected cells | | | |
| --- | --- | --- | --- |
| **Protein** | **Average fold change** | **SD or**  **(SEM)** | **No. of experiments** |
| p110α | +14 | (0.37) | 2 |
| p85α | +18 | (0.95) | 2 |
| p85β | +10 | (3.0) | 2 |
| P-Akt(S473) | +21 | 8.5 | 4 |
| P-Akt(T308) | +25 | N/A | 1 |
| Akt | +3.6 | 2.3 | 4 |
| Dlg1 | -2.6 | (0.32) | 2 |
