# Supplementary material for: The Human Adenovirus E4-ORF1 Protein Subverts Discs Large 1 to Mediate Membrane Recruitment and Dysregulation of Phosphatidylinositol 3-Kinase
Source: PLoS Pathog. 2014 May 1;10(5):e1004102. doi: 10.1371/journal.ppat.1004102 (PMC4006922; doi:10.1371/journal.ppat.1004102)
Supplement: Table S8 — Average fold changes in protein levels quantified from immunoblots of Ad9-V125A virus- versus mock-infected cells. For Figure 6A, average fold changes in levels of the indicated proteins were quantified from independent immunoblots of Ad9-V125A virus-infected cells versus mock-infected cells. See Materials and Methods for details. (DOCX) [file ppat.1004102.s011.docx]

| **Table S8.**  Average fold changes in protein levels quantified from immunoblots of Ad9‑V125A virus- *versus* mock-infected cells | | | |
| --- | --- | --- | --- |
| **Protein** | **Average fold change** | **SD or**  **(SEM)** | **No. of experiments** |
| p110α | +13 | (0.49) | 2 |
| p85α | +18 | (0.82) | 2 |
| p85β | +3.9 | (0.15) | 2 |
| P-Akt(S473) | +2.7 | 1.22 | 4 |
| P-Akt(T308) | +2.8 | N/A | 1 |
| Akt | +2.8 | 1.65 | 4 |
| Dlg1 | -2.8 | (0.08) | 2 |
