# Supplementary material for: The Human Adenovirus E4-ORF1 Protein Subverts Discs Large 1 to Mediate Membrane Recruitment and Dysregulation of Phosphatidylinositol 3-Kinase
Source: PLoS Pathog. 2014 May 1;10(5):e1004102. doi: 10.1371/journal.ppat.1004102 (PMC4006922; doi:10.1371/journal.ppat.1004102)
Supplement: Table S9 — Average percentage of vector cells versus wt ORF1 cells showing E4-ORF1, p85, P-Akt, and Akt protein staining at the plasma membrane in IF assays. For Figures 8, 9, 11, and S3, the average percentages of vector cells versus wtORF1 cells exhibiting plasma membrane staining for the indicated proteins were quantified from independent IF assays. See Materials and Methods for details. (DOCX) [file ppat.1004102.s012.docx]

| **Table S9.** Average percentage of vector cells *versus* *wt*ORF1 cells showing E4-ORF1, p85, P‑Akt, and Akt protein staining at the plasma membrane in IF assays | | | | | | | |
| --- | --- | --- | --- | --- | --- | --- | --- |
| **Protein** | **vector cells** | | | ***wt*ORF1 cells** | | | ***p*-value** |
|  | **% cells with plasma membrane staining ± SD (SEM)** | **No. of cells** | **No. of expts** | **% cells with plasma membrane staining ± SD** | **No. of cells** | **No. of expts** |  |
| E4-ORF1 | 0.0% ± 0.0% | 608 | 3 | 97% ± 0.60% | 644 | 3 | 9.7E-10*** |
| p85 | 0.88% ± 0.93% | 462 | 3 | 93% ± 2.2% | 407 | 3 | 3.4E-07*** |
| P-Akt (S473) | 0.13% ± 0.23% | 658 | 3 | 97% ± 1.7% | 676 | 3 | 6.3E-08*** |
| Akt | 6.4% ± (4.6%) | 395 | 2 | 99% ± 1.4% | 671 | 3 | N/A |
